# Socioeconomic disparities and household crowding in association with the fecal microbiome of school-age children

**DOI:** 10.1038/s41522-022-00271-6

**Published:** 2022-03-03

**Authors:** Yelena Lapidot, Leah Reshef, Mayan Maya, Dani Cohen, Uri Gophna, Khitam Muhsen

**Affiliations:** 1 Department of Epidemiology and Preventive Medicine, School of Public Health, The Sackler Faculty of Medicine, Tel Aviv University, Ramat Aviv, Tel Aviv, 6139001 Israel; 2 The Shmunis School of Biomedicine and Cancer Research, Faculty of Life Sciences, Tel Aviv University, Tel Aviv, Israel

**Keywords:** Microbiota, Health care

## Abstract

The development of the gut microbiome occurs mainly during the first years of life; however, little is known on the role of environmental and socioeconomic exposures, particularly within the household, in shaping the microbial ecology through childhood. We characterized differences in the gut microbiome of school-age healthy children, in association with socioeconomic disparities and household crowding. Stool samples were analyzed from 176 Israeli Arab children aged six to nine years from three villages of different socioeconomic status (SES). Sociodemographic data were collected through interviews with the mothers. We used 16 S rRNA gene sequencing to characterize the gut microbiome, including an inferred analysis of metabolic pathways. Differential analysis was performed using the analysis of the composition of microbiomes (ANCOM), with adjustment for covariates. An analysis of inferred metagenome functions was performed implementing PICRUSt2. Gut microbiome composition differed across the villages, with the largest difference attributed to socioeconomic disparities, with household crowding index being a significant explanatory variable. Living in a low SES village and high household crowding were associated with increased bacterial richness and compositional differences, including an over-representation of *Prevotella copri* and depleted *Bifidobacterium*. Secondary bile acid synthesis, d-glutamine and d-glutamate metabolism and Biotin metabolism were decreased in the lower SES village. In summary, residential SES is a strong determinant of the gut microbiome in healthy school-age children, mediated by household crowding and characterized by increased bacterial richness and substantial taxonomic and metabolic differences. Further research is necessary to explore possible implications of SES-related microbiome differences on children’s health and development.

## Introduction

The colonization of the human gut with microorganisms begins at birth and is characterized by a succession of microbial consortia, which is influenced by changes in diet and life events^1^. During the first few years of life, the gut microbiome gradually develops its structure and function, driven by genetic disposition and environmental exposures. In contrast to the common belief that the gut microbiome approaches adult (and relatively stable) levels in early childhood^2^, recent findings demonstrated substantial functional and taxonomic bacterial differences in the gut microbiota of healthy children with respect to those of adults, suggesting that the gut microbiome may develop more slowly than previously thought^3–8^.

While the association between early life determinants and the development of the gut microbiome in infancy has been investigated, the potential persistent influence of environmental factors on the gut microbial community at later childhood remains largely unknown^9^. Moreover, recent cross-sectional studies highlighted the importance of environmental features in shaping the microbiome throughout the life cycle^10–13^. Individual-level determinants of the microbiome are not necessarily identical to those that explain differences across populations, especially those living within homogeneous environments with respect to geography, culture, and nutrition^14^. Social contexts including living conditions, income, and education, are emerging both as fundamental determinants of the microbiome^15^ and as possible modifiers of the existing microbiome–health associations across the life course^16^. A better understanding of the separate influences of socioeconomic indicators on the gut microbiome is essential given the growing evidence on the importance of the microbiome in human health and disease, and the role of social determinants in health disparities.

Emerging socioeconomic indicators in the context of the microbiome include socioeconomic status (SES) and household composition. A recent review^14^ identified two studies that linked various socioeconomic conditions to differences in the gut microbiome^13,17^. Bowyer et al.^13^, showed associations between individual and area-level income to the gut microbial diversity and composition, while Miller et al.^17^ showed a higher α-diversity with increased neighborhood SES. Lane et al.^18^, revealed that household composition of breastfed infants is associated with variation in the gut microbiome, suggesting that a higher diversity of cohabitants may facilitate social bacterial transmission to the infant’s gastrointestinal tract, via shared environment or direct physical and social contact between the maternal-infant dyad and other household members.

These findings demonstrate the influence of the social environment on the gut microbiome; however, evidence on this association during childhood years remains elusive. Childhood is a crucial period for physical and cognitive development^19^, thus environmental exposures during this period may have a key role in the child’s health and wellbeing as an adult. Our study aimed to explore the relationships between individual-level and residential SES indicators and the fecal microbiome diversity and composition of healthy school-age children from villages of different SES. The study was conducted in three Arab villages located in the same geographic region in northern Israel. We hypothesized that despite shared ethnicity, geographic location and cultural dietary habits, SES disparities across the villages, and particularly the household crowding, will be associated with microbial differences.

## Results

### Demographic characteristics of the participants

Overall 192 children provided fecal specimens, of these 186 (96.9%) had a sufficient amount of fecal material for genomic DNA extraction. After initial quality control, 176 (91.7%) were included in the analysis. The participants’ age ranged from 6.0 to 9.9 years (mean = 7.7, SD = 0.9 years), 40.3% of the participants were females. The household crowding index ranged from 0.6 to 7.0 (mean 1.9 [SD = 1.1]). The study included 47 (26.7%) children from village A, 59 (33.5%) from village B, and 70 children (39.8%) from village C (Table 1). Significant differences were found between the villages in socioeconomic factors (Table 1). The mean household crowding index was significantly higher in village C compared to village B (*p* < 0.001), and village A (*p* < 0.001), but no significant difference was found between villages A and B (*p* = 0.825; Supplementary Fig. 1a). The median number of siblings was significantly higher in village C compared to the other two villages (*p* < 0.001 for both comparisons). Household monthly income was significantly lower in village C compared to villages B (*p* = 0.03), and A (*p* < 0.001), but no significant difference was found between villages A and B (*p* = 0.157). The mean number of maternal schooling years was lower in village C compared to the other two villages (*p* < 0.001 for both comparisons), but no significant difference was found between villages A and B (*p* = 0.550; Supplementary Fig. 1b). There were significant differences in the mean body mass index (BMI) Z score of children from villages A and B (*p* < 0.001). There were no significant differences between the villages in sex distribution, breastfeeding in infancy (Supplementary Fig. 2a) and daycare attendance in early life (Table 1). Given the differences between village C compared to villages A and B, in the subsequent analyses we combined data of participants from villages A and B that represented the intermediate/higher SES villages, while village C represented low SES village.

**Table 1:** Demographic characteristics of the study participants by village of residence.

	Village A *N* = 47	Village B *N* = 59	Village C *N* = 70	*p*-value
Age, years, mean (SD)	8.6 (0.6)	7.2 (0.6)	7.5 (0.8)	<0.001^d^
Sex, females, *N* (%)	19 (40.4%)	21 (35.6%)	31 (44.3%)	0.604
Household crowding index^a^, mean (SD)	1.3 (0.5)	1.6 (0.7)	2.5 (1.3)	<0.001^e^
Household income^b^, *N* (%)				<0.001^f^
Above average	10 (28.6%)	5 (8.8%)	2 (2.9%)	
Average	11 (31.4%)	17 (29.8%)	19 (27.5%)	
Below average	4 (11.4%)	18 (31.6%)	11 (15.9%)	
Much below average	10 (28.6%)	17 (29.8%)	37 (53.6%)	
Number of siblings, median (IQR)	3 (2)	2 (1.5)	4 (3)	<0.001^g^
Father’s schooling years, mean (SD)	11.8 (3.7)	10.6 (3)	8.6 (3.3)	<0.001^h^
Mother’s schooling years, mean (SD)	11.7 (3.7)	10.7 (3.4)	6.5 (3.8)	<0.001^i^
BMIZ score^c^, mean (SD)	0.37 (1.0)	0.02 (1.1)	0.82 (0.9)	<0.001^j^
Breastfeeding, yes, *N* (%)	45 (95.7%)	57 (96.6%)	61 (87.1%)	0.08
Age (months) of introducing solid foods, mean (SD)	6.1 (2.9)	5.9 (2.9)	5.8 (2.6)	0.827
Daycare center in early life, *N* (%)	11 (23.4%)	10 (17.0%)	13 (18.6%)	0.697

*BMIZ* body mass index Z score, *IQR* interquartile range, *SD* standard deviation.

^a^Household crowding: Number of people living in the household/Number of rooms in the household.

^b^Household income: Household income as compared to the national average.

^c^Participants missing BMIZ scores (*n* = 4) imputed with the median value of the cohort.

^d^Post-hoc pairwise comparison resulted in significant difference between all 3 villages in the mean age.

^e^Post-hoc pairwise comparisons: village C vs. village A (*p* < 0.001); village C vs. village B (*p* < 0.001); and village B vs. village A *p* = 0.825.

^f^Post-hoc pairwise comparisons: village C vs. village A (*p* < 0.001); village C vs. village B (*p* = 0.03); and village B vs. village A (*p* = 0.157).

^g^Post-hoc pairwise comparisons: village C vs. village A (*p* < 0.001); village C vs. village B (*p* < 0.001); and village B vs. village A (*p* = 0.9).

^h,i^Post-hoc pairwise comparison: the mean father’s and mother’s schooling years village C vs. village B *p* = 0.002; *p* < 0.001, respectively); village C vs. village A (*p* < 0.001 for both), and village B vs. village A (*p* = 0.163; *p* = 0.550, respectively).

^j^Post-hoc pairwise comparisons: village C vs. village B (*p* < 0.001), no significant difference between the other villages.

Qualitative information on diet was collected at age three to five years for 92 participants via maternal interviews. There were no significant differences in the consumption of vegetables (*p* = 0.06), fruit (*p* = 0.06), red meat (*p* = 0.532), and poultry (*p* = 0.601) by village of residence, with the majority of participants reporting regular consumption of the aforementioned food groups (Supplementary Fig. 2b–e).

### Bacterial diversity and composition across villages

**Fig. 1:**
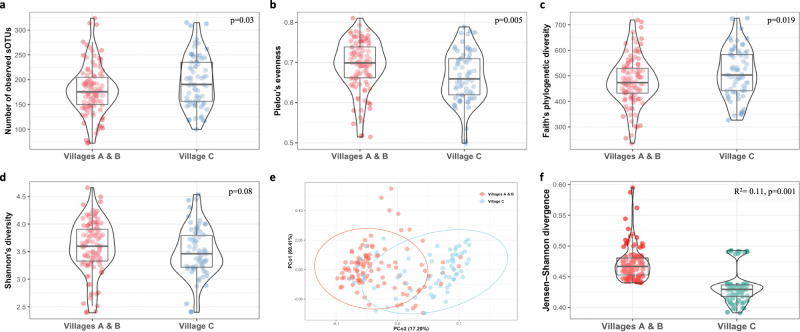
Divergent socioeconomic villages and the gut microbiome diversity and composition. **a** Box-violin plots of microbial richness, measured by the number of observed s-OTUs. **b** Box-violin plots of microbial α-diversity, measured by Pielou’s evenness index. **c** Box-violin plots of microbial α-diversity, measured by the phylogenetic-based metric Faith’s PD. **d** Box-violin plots of microbial α-diversity, measured by the Shannon’s diversity index. *P*-value for the differences between villages A and B vs. village C: *p* = 0.005 for Pielou’s evenness index, *p* = 0.03 for the number of observed s-OTUs, *p* = 0.019 for Faith’s PD, and *p* = 0.08 for Shannon’s diversity. **e** Principal coordinate analysis of the JSD notably different among children villages A and B vs. village C (PERMANOVA R^2^ = 0.11, *p* = 0.001). **f** Box-violin plots of the mean JSD distances across villages. Within the study population, villages A and B represent the intermediate/high SES villages, while village C represents the lower SES village. The sample size in each village was 47, 59, and 70, respectively. In the box-violin plots (panels **a, b, c, d, f**), the centre line represents the median, the lower bound of the box represents the 25th percentile, the upper bound of the box represents the 75th percentile, the lowest point of the lower whisker represents the minimum value and the highest point of the upper whisker represents the maximum value. The violin plot implements a rotated kernel density plot on each side, adding information regarding the full distribution of the measured data; the width of the violin indicates the frequency.

Bacterial α-diversity was significantly different in participants from village C compared to those from villages A and B, measured by the higher number of observed “sub” operational taxonomic units (s-OTUs) (*p* = 0.03; Fig. 1a), the decreased Pielou’s evenness index (*p* = 0.005; Fig. 1b) and increased Faith’s PD index (*p* = 0.019; Fig. 1c). Shannon’s diversity index was decreased in the lower SES compared to the higher SES villages, but this association was not statistically significant (*p* = 0.08; Fig. 1d). There were significant differences in both the dispersion and centroids between the villages. Beta dispersion test was significant (F = 7.4, *p* = 0.01) and the fecal bacterial composition was different, as measured by the Jensen-Shannon divergence (JSD) index (R^2^ = 0.11, *p* = 0.001) and by the Weighted UniFrac distance (R^2^ = 0.107, *p* = 0.001), adjusted for sex, age, and household crowding index (Fig. 1e, f). Sex and age were not significantly associated with the microbial differences; however, a significant difference was found according to household crowding (JSD R^2^ = 0.018, *p* = 0.002; Weighted UniFrac R^2^ = 0.015, *p* = 0.004) (Supplementary Table 1a and b). The fully adjusted model resulted in similar significant composition differences and highest variance attributed to the diverging SES villages (JSD R^2^ = 0.113, *p* = 0.001; Weighted UniFrac distance R^2^ = 0.104, *p* = 0.001) (Supplementary Table 2a and b). Crowding index remained a significant covariate explaining a large amount of variance in the fully adjusted model based on both the JSD and the Weighted UniFrac distance (R^2^ = 0.018, *p* = 0.004 and R^2^ = 0.015, *p* = 0.006, respectively).

### Taxonomic differences across villages

**Fig. 2:**
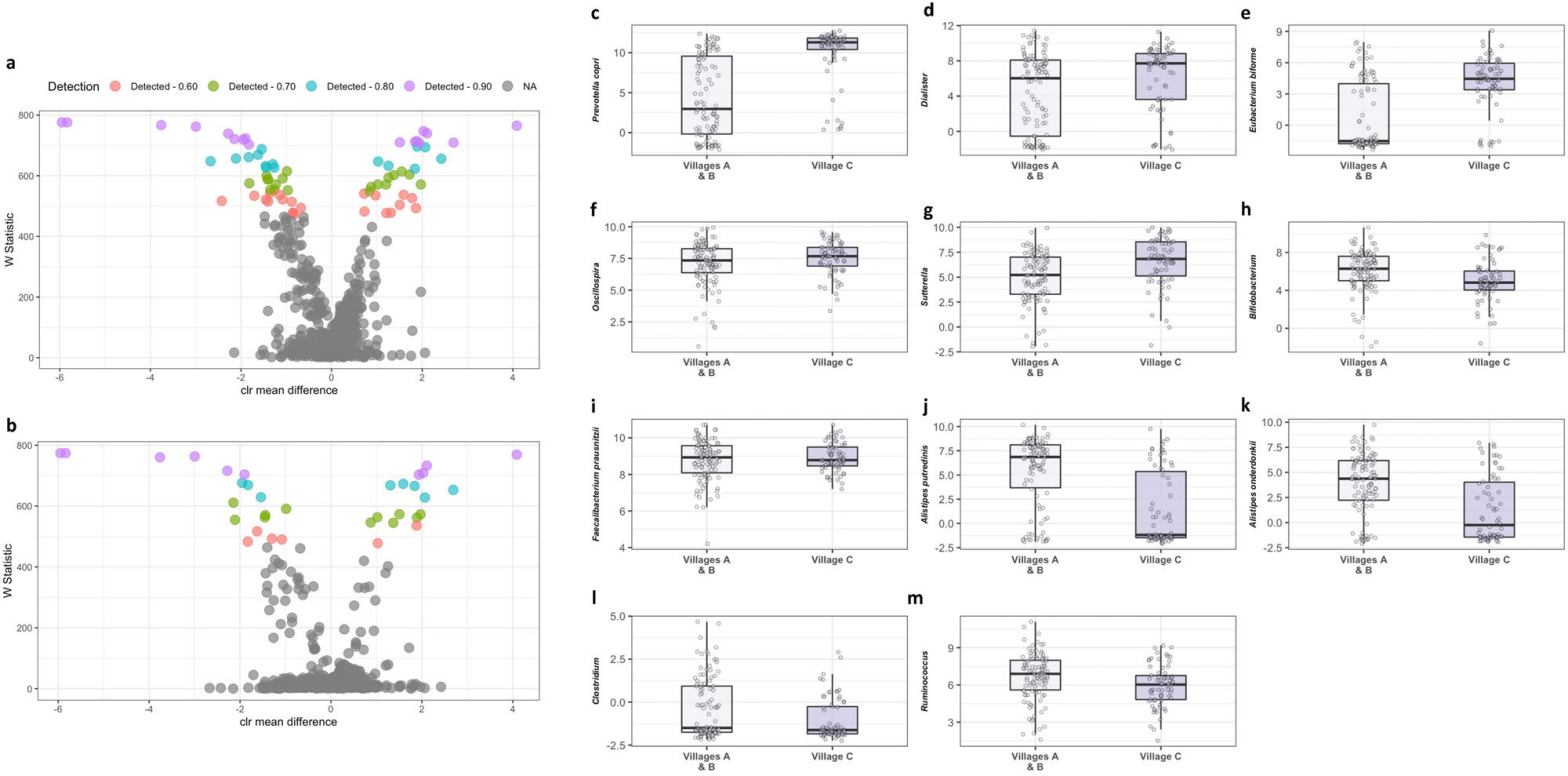
Differentially abundant taxa by village of residence and socioeconomic status. **a, b** Volcano plots showing differentially abundant s-OTUs as detected by ANCOM (partially adjusted model (**a**) and the fully adjusted model (**b**). The *x*-axis represents the difference in mean centered log ratio (clr)-transformed abundance between groups and the *y*-axis represents the ANCOM W Statistic. s-OTU points are colored by level of ANCOM significance, with 0.9 being the highest level; s-OTUs in gray were not significant. **c–m** Boxplots of clr-transformed abundance for s-OTUs detected by ANCOM at the highest level (0.9) in association to the village of residence and socioeconomic status. The relative abundance of *Prevotella copri*, *Dialister, Eubacterium biforme, Oscillospira*, and *Sutterella* was increased in village C compared to villages A and B, while *Bifidobacterium*, *Faecalibacterium prausnitzii, Alistipes putredinis*, *Alistipes onderdonkii*, *Clostridium*, and *Ruminococcus* were depleted. In the boxplots (panels **c–m**), the centre line represents the median, the lower bound of the box represents the 25th percentile, the upper bound of the box represents the 75th percentile, the lowest point of the lower whisker represents the minimum value and the highest point of the upper whisker represents the maximum value. Within the study population, villages A and B represent the intermediate/high SES villages, while village C represents the lower SES village. The sample size in each village was 47, 59, and 70, respectively.

An analysis of the composition of microbiomes (ANCOM) comparing the fecal microbiome of children from village C to those from villages A and B resulted in 72 significant features discriminating the villages, adjusted for age, sex, and household crowding (i.e., reduced model; Fig. 2a, Supplementary Table 3). At the highest detection level of 0.9, we observed significantly increased abundances of the family Ruminococcaceae and the genera *Prevotella copri*, *Dialister, Eubacterium biforme*, Ruminococcaceae *Oscillospira* and *Sutterella*, and depleted abundance of *Bifidobacterium*, *Faecalibacterium prausnitzii, Alistipes putredinis*, *Alistipes onderdonkii*, *Clostridium*, and *Ruminococcus* in village C compared to villages A and B (Fig. 2c−m). At detection level 0.8, we observed decreased abundances of *Bacteroides ovatus*, *Bacteroides uniformis*, *Parabacteroides distasonis* and an over-representation of Prevotellaceae, including *Prevotella stercorea*.

The fully adjusted model detected similar taxa at the detection level of 0.9 (Fig. 2b, Supplementary Table 4), except *Sutterella, Alistipes onderdonkii*, *Clostridium,* and Ruminococcaceae, which were detected at a lower level of 0.8. *Bacteroides ovatus*, Mogibacteriaceae were unaffected by the adjustments. *Parabacteroides distasonis*, Prevotella, were detected at 0.7 compared to 0.8 in the reduced model, whereas no significant difference was found in *Bacteroides uniformis, Catenibacterium*, and *Prevotella stercorea* in the fully adjusted model.

### Household crowding and microbial diversity

**Fig. 3:**
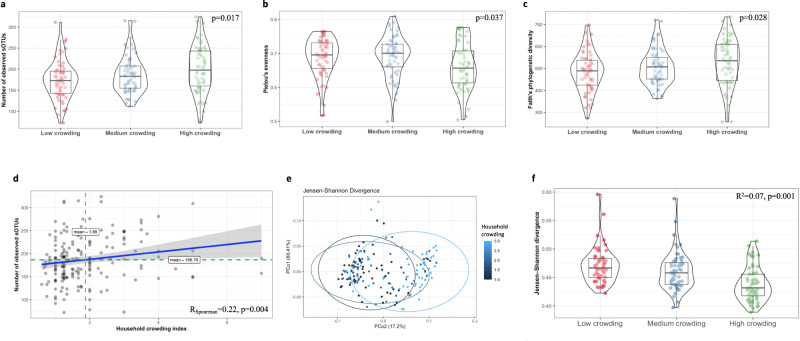
Household crowding, gut microbiome diversity and composition. **a** Box-violin plots of microbial richness measured by the number of observed s-OTUs. **b** Box-violin plots of microbial α-diversity measured by Pielou’s evenness index. **c** Box-violin plots of microbial α-diversity, measured by the phylogenetic-based metric Faith’s PD. *P*-value for the difference in fecal α-diversity according to household crowding, as measured by the number of observed s-OTUs (*p* = 0.017), Pielou’s evenness index (*p* = 0.037), and *p* = 0.028 for Faith’s PD. Pairwise comparisons: The number of observed s-OTUs: lowest vs. highest tertiles *p* = 0.024. Pielou’s evenness index: the lowest vs. middle tertiles and the middle vs. highest tertile (p = 0.039 for both comparisons), and Faith’s PD lowest vs. highest tertiles *p* = 0.02. **d** Non-parametric Spearman’s correlation coefficient *r* = 0.22, *p* = 0.004) of the correlation between household crowding index and bacterial richness. **e** Principal coordinate analysis of the JSD notably different across fluctuating household crowding tertiles (*p* = 0.001). **f** Box-violin plots of the mean JSD distances in association with household crowding, significantly different with dissimilarities in household crowding (lowest vs. middle tertile (*p* = 0.001) and lowest vs. highest tertiles (*p* < 0.001). Household crowding index was calculated as the number of people living in the household by the number of rooms in the household. The variable was categorized by tertiles. In the box-violin plots (panles **a, b, c, f**), the centre line represents the median value, the lower bound of the box represents the 25th percentile, the upper bound of the box represents the 75th percentile, the lowest point of the lower whisker represents the minimum value and the highest point of the upper whisker represents the maximum value. The violin plot implements a rotated kernel density plot on each side, adding information regarding the full distribution of the measured data; the width of the violin indicates the frequency.

There was a strong association between household crowding and fecal α-diversity, as measured by the number of observed s-OTUs (*p* = 0.017, Fig. 3a), Pielou’s evenness index (*p* = 0.037, Fig. 3b), and increased Faith’s PD index (*p* = 0.028, Fig. 3c). Pairwise comparisons of household crowding index (represented as tertiles), resulted in substantial differences between the lower and highest tertiles, as measured by the number of observed s-OTUs (*p* = 0.024). Pielou’s evenness index differed significantly between the lowest vs. middle tertiles and between the middle vs. highest tertile (*p* = 0.039 for both comparisons). Similarly, there was a positive correlation between bacterial richness and household crowding index (Spearman’s *r* coefficient = 0.22, *p* = 0.004) (Fig. 3d).

We found significant compositional differences of the fecal microbiome in association to crowding index, as measured by the JSD (R^2^ = 0.07, *p* = 0.001; Fig. 3e). There was a significant difference in bacterial dispersion between the lowest tertile of crowding index compared to middle and highest household crowding tertiles, suggesting a less variable microbiome with increased household crowding (*p* < 0.001) (Fig. 3f). The model showed a significant association between the participants’ age and village with the microbial composition (Supplementary Table 5A); however, most variance was explained by crowding index (F = 14.4, R^2^ = 0.072), followed by village of residence (F = 6.6, R^2^ = 0.065), while age explained less variance (F = 2.2, R^2^ = 0.011) (Supplementary Table 5A). The analysis of the phylogenetic Weighted UniFrac distance showed a similar trend of significant bacterial differences between the lowest compared to middle and highest household crowding tertiles (R^2^ = 0.052, *p* = 0.001). Additionally, associations were found with the participants’ age (R^2^ = 0.012, *p* = 0.01) and village of residence (R^2^ = 0.047, *p* = 0.001) (Supplementary Table 5B).

### Taxonomic differences associated with household crowding index

**Fig. 4:**
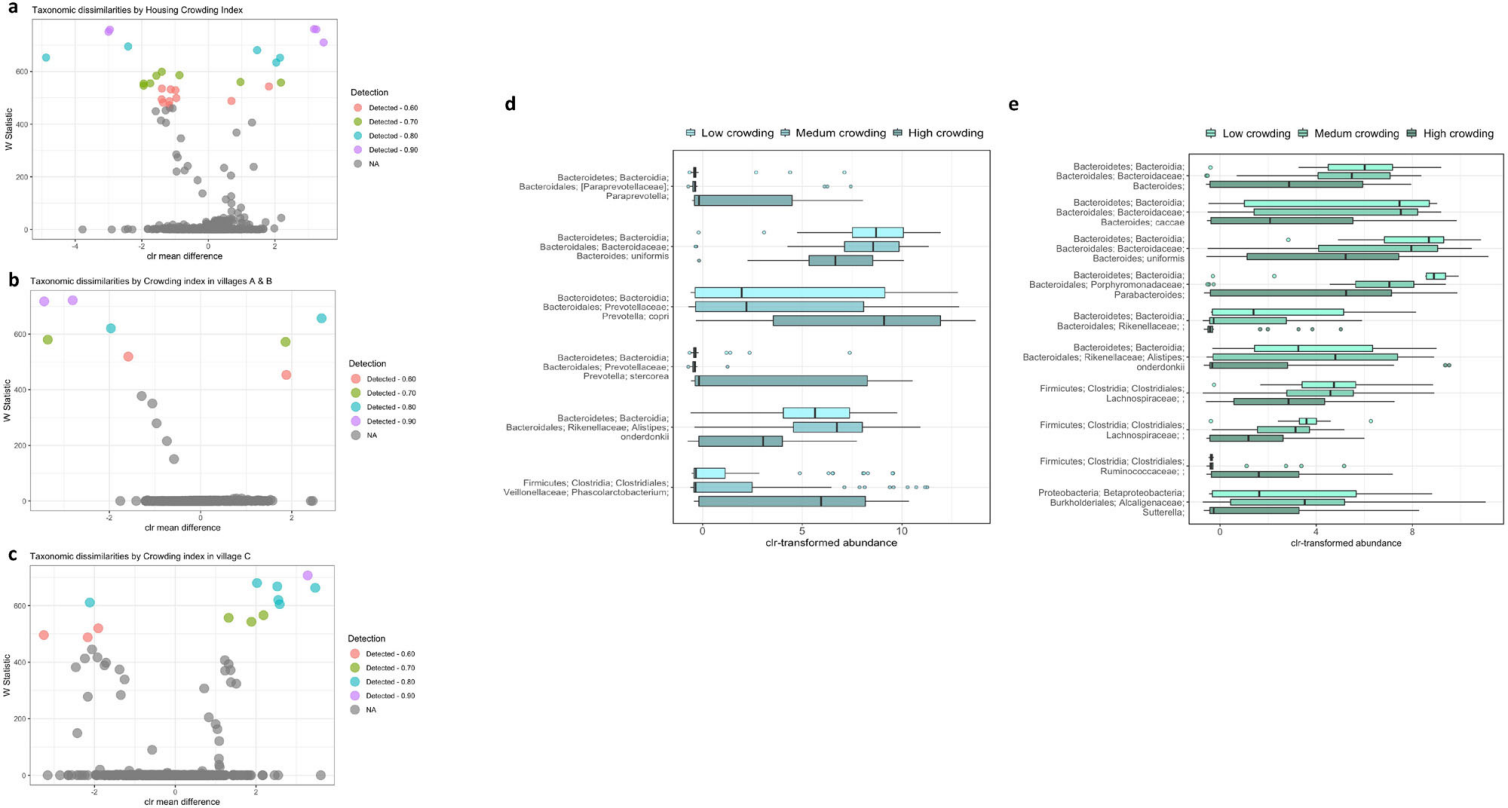
Differentially abundant taxa by village of residence and socioeconomic status. **a–c** Volcano plots showing differentially abundant s-OTUs as detected by ANCOM, in all three study villages (**a**) and stratified by village; villages A and B [higher SES] (**b**), and village C [low SES] (**c**). The *x*-axis represents the difference in mean centered log ratio (clr)-transformed abundance between groups and the *y*-axis represents the ANCOM W Statistic. s-OTU points are colored by level of ANCOM significance, with 0.9 being the highest level; s-OTUs in gray were not significant. **d, e** Boxplots of clr-transformed abundance of s-OTUs significantly associated with the household crowding index in villages A and B [higher SES] (**d**) and in village C [low SES] (**e**), adjusted for sex, age, and village of residence. Tertiles of crowding index were categorized as low, middle and high household crowding tertiles. In the boxplots (panles **d, e**), the centre line represents the median, the lower bound of the box represents the 25th percentile, the upper bound of the box represents the 75th percentile, the lowest point of the lower whisker represents the minimum value and the highest point of the upper whisker represents the maximum value. Within the study population, villages A and B represent the intermediate/high SES villages, while village C represents the lower SES village. The sample size in each village was 47, 59, and 70, respectively.

An ANCOM analysis, adjusted for age, sex, and village, resulted in significantly differentially abundant bacteria associated with household crowding index (Fig. 4a). At the highest detection level, we observed different relative abundance of *Alistipes onderdonkii*, *Bacteroides uniformis*, *Prevotella stercorea*, *Phascolarctobacterium,* and *Alistipes putredinis* at detection level 0.9, and *Faecalibacterium prausnitzii*, Lachnospiraceae, *Prevotella copri*, *Parabacteroides*, and Bacteroides at detection level 0.8 (Supplementary Table 6). A stratified analysis by village showed significant compositional differences associated with household crowding in children from villages A and B and village C (Fig. 4b, c; Supplementary Table 7). Interestingly, the relative abundance of *Bacteroides uniformis* and *Alistipes onderdonkii* was inversely associated with household crowding index in all villages. In the villages A and B higher household crowding was associated with an increased abundance of *Prevotella stercorea*, *Paraprevotella*, *Prevotella copri,* and *Phascolarctobacterium* (Fig. 4d). Moreover, in village C, an increase in household crowding index was associated with an over-representation of Ruminococcaceae, while *Bacteroides caccae, Parabacteroides*, Lachnospiraceae, and *Sutterella* were depleted (Fig. 4e).

### Village SES, household crowding, and inferred metabolic pathways

**Fig. 5:**
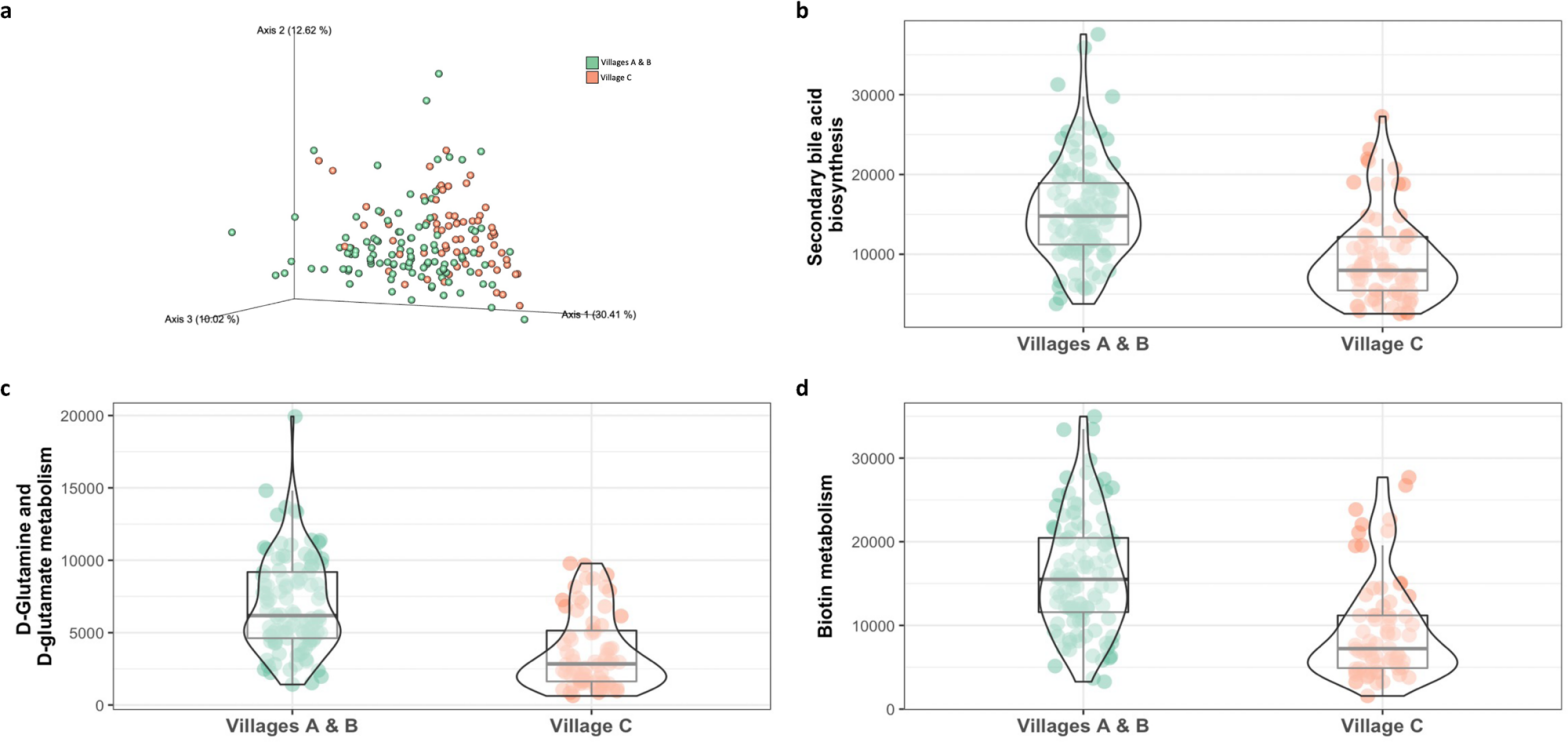
Differentially abundant inferred metabolic functions by village of residence and socioeconomic status. **a** Principal coordinate analysis of the Bray-Curtis dissimilarity notably different among children from villages A and B vs. village C (*p* = 0.001). **b–d** Differentially abundant inferred metabolic pathways detected by ANCOM in association with village (SES) and with household crowding. Within the study population, villages A and B represent the intermediate/high SES villages, while village C represents the lower SES village. The sample size in each village was 47, 59, and 70, respectively. In the box-violin plots (panles **b–d**), the centre line represents the median value, the lower bound of the box represents the 25th percentile, the upper bound of the box represents the 75th percentile, the lowest point of the lower whisker represents the minimum value and the highest point of the upper whisker represents the maximum value. The violin plot implements a rotated kernel density plot on each side, adding information regarding the full distribution of the measured data; the width of the violin indicates the frequency.

An analysis of metabolic pathways inferred from bacterial 16 S rRNA marker gene resulted in significantly different composition (Bray-Curtis dissimilarity distance *p* = 0.001; Fig. 5a). We then performed an exploratory ANCOM analysis, that detected differentially abundant pathways between village C compared to villages A and B, including increased d-glutamine and d-glutamate metabolism (W = 208), secondary bile acid biosynthesis (W = 188), and Biotin metabolism (W = 188) in villages A and B compared to village C (Fig. 5b–d).

### Sensitivity analyses

#### Differences in microbial β-diversity

Since Beta dispersion test was statistically significant, we performed ANOSIM (analysis of similarities) test, to further confirm our findings regarding microbial β-diversity. Consistent with the permutational multivariate analysis of variance (PERMANOVA) results, ANOSIM test showed significant difference between the study villages (r^2^ = 0.28, *p* = 0.001) and according to household crowding (r^2^ = 0.064, *p* = 0.016). The null hypothesis can be rejected for both comparisons; the magnitude of the difference was stronger by village of residence than household crowding conditions. Collectively these results show statistically significant differences in the microbial communities between the study groups.

#### Re-analysis using a random sub-sampling approach

Since the sample size differed across the study villages, we reanalysed the data using random and equal subsamples in the three villages, so each village included 47 random samples (the lowest sample size). The sociodemographic characteristics of the subsamples were similar to the original study groups, and the significant SES differences between the villages were also maintained in the subsamples (Supplementary Fig. 3). The analysis addressing the associations of the village of residence and household crowding with microbial associations (alpha diversity, microbial composition, and taxonomic differences) using the subsamples showed consistent results with findings obtained using the entire study sample and original groups, despite the smaller sample sizes (Supplementary Figs. 4–9, Supplementary Tables 8–11).

#### Antibiotic use and diarrheal diseases

Information was obtained on antibiotic use during the 6 months preceding enrollment and sample collection and was available for 163 (92.6%) participants, which was reported among 54% of them. There were no significant differences in bacterial alpha diversity according to antibiotic use (Supplementary Fig. 10). We added the variable antibiotic use as a covariate to our model of Weighted UniFrac, which showed a nonsignificant association (R^2^ = 0.008, *p* = 0.148). The JSD detected significant differences (R^2^ = 0.01, *p* = 0.031); however, the effect was weaker compared to the village of residence and the household crowding. Differential abundance analysis using ANCOM revealed small differences including Erysipelotrichaceae, *Coprococcus,* and Clostridiales. These taxonomic differences were significantly more delicate compared to the taxonomic differential abundance analysis in association with village of residence and household crowding, which, in line with the high effect size for the analysis of the latter variables (Supplementary Fig. 10g).

No significant differences were found between diarrheal diseases in the past year and intestinal microbiome (Supplementary Fig. 11a–c).

## Discussion

We examined the relationships between socioeconomic disparities, household crowding index and the gut microbiome of healthy school-age children. We demonstrated that the environmental and social exposures are notably associated with differences in gut microbial ecology, despite the shared ethnicity, cultural, dietary, and geographic characteristics of this cohort. Moreover, we showed significant associations between environmental exposures and children’s intestinal microbiome during an important period for physical growth and development.

We demonstrated that both residential and household SES, including household living crowding conditions, are related to the gut microbiome of healthy school-age children. The results were consistent using multiple analytical methods, both when analyzing the full dataset and using random subsamples of the study groups, thus strengthening our original findings that lower SES and higher household crowding are significantly associated with increased alpha diversity, different bacterial composition and taxonomic variations among school-age children. Additionally, our model reduces possible confounders, since all participants were from the same ethnicity, live in the same geographic location and share the same health care system. Membership of an ethnic group defines shared cultural heritage, including cuisine and dietary habits, lifestyle and practices of childcare. For example, in the current cohort, breastfeeding was highly common, and the utilization of daycare was relatively low and occurred late. Beyond SES variation and household crowding conditions that play an important role in shaping the gut microbiome, within-population differences in the consumption quality of macro and micronutrients favorable for the gut microbiota likely contribute to the observed microbial-SES differences in our study population.

We observed a significant association between the intestinal bacterial diversity amongst the villages. There was a significant increase in fecal bacterial richness, increased phylogenetic diversity, and complementary depleted microbial evenness amid participants from a lower SES village (village C). We found a similar trend with an increased household crowding index, a measurement that is highly correlated with SES. This unique observation can be linked to Rook’s “old friends” hypothesis^20^, a revised version of Strachan’s “hygiene hypothesis”^21^, proposing that larger family size, and having siblings might confer benefits to the immune system, possibly via the modulation of the microbiome. An alternative hypothesis might be that several bacterial taxa require extended contact to invade family members. This could explain the profound increase in bacterial richness combined with depleted evenness, suggesting dominant colonization of selected bacterial species. These species could be associated with higher household crowding and thus, extended contact with more family members. Nevertheless, currently there are significant gaps in understanding of how the household and social environments differentially facilitate the horizontal transmission of microbiota in children, emphasizing the need for large-scale longitudinal studies.

We demonstrate that a higher household crowding index, which reflects exposure to caregivers and siblings, is associated with higher microbial diversity and richness. Interestingly, associations were demonstrated between various diseases affecting westernized countries with dysbiosis and loss of microbial diversity in the gut microbiota^22^. This cohort focuses on healthy children, thus a significant decrease in microbial biodiversity was not expected. Higher household crowding index is closely associated with lower SES, which is in turn linked to lifestyle patterns and health outcomes in adulthood^23–25^. As such, SES disparities, traditionally measured through education, income, and/or occupation, are considered as the most fundamental cause of health disparities^25,26^. Since lower SES is often correlated with excess morbidity and mortality worldwide and locally in Israel^27,28^, and simultaneously strongly associated with microbial differences^13,17^, large-scale longitudinal studies are needed to explore the development of the child’s microbiome, including the possible fluctuations of bacterial diversity and long term health-related outcomes.

We also observed differences in microbial composition associated with SES, detected both by the Weighted UniFrac and by the JSD and consistent by the implementation of both PERMANOVA and ANOSIM. Moreover, it appears that the low SES village (village C) was the most divergent compared to the higher SES villages. The results were similar in a fully adjusted permutation-based analysis of the variance model. Our fully adjusted PERMANOVA model explained 14.7% (JSD) of the microbial variation in this cohort, with the village of residence explaining the largest amount of variance (11.4%), followed by household crowding. The examined covariates had significant associations, albeit they explained a relatively low percentage of variance in this cohort. Notably, although the village of residence and household crowding retained the strongest effect, still they did not account for all the variance, suggesting additional contributors from unidentified influences, stochastic effects, or biotic interactions. These findings are in line with updated literature, emphasizing the gap in the understanding of microbiome variance and the need for further exploration of unknown covariates as well as intrinsic microbial ecological processes such as founder effects, species interactions, and dynamics^29^.

The village of residence was also associated with taxonomic differences, after partial and full adjustment for potential confounders. The strongest consistent effect was observed for *Prevotella copri*, *Alistipes putredinis*, *Eubacterium biforme*, *Dialister*, *Faecalibacterium prausnitzii*, *Bifidobacterium*, *Oscillospira*, *Ruminococcus,* and *Sutterella*. *Prevotella copri* is considered an important yet enigmatic member of the gut microbiome, being a common human gut microbe that has been both positively and negatively associated with human health, including an association with inflammatory diseases^30–32^, insulin resistance and glucose intolerance^33^. While other studies linked between *Prevotella copri* and improved glucose and insulin tolerance in association with dietary fiber^34,35^, suggesting that the beneficial effects could be diet-dependent. Additionally, a higher prevalence of xylan-degrading *Prevotella*^36^ has been consistently reported in non-Westernized populations^37,38^ that typically consume diets rich in fresh, plant-based and unprocessed food. Indeed, although Westernization encompasses more factors and lifestyle modifications than diet alone, *Prevotella copri* was strongly associated with consumption of high fiber, low fat and low animal protein diets than typical Western diets^39,40^.

Interestingly, the microbial signature found in village C included taxonomic differences in line with global studies of rural communities^41,42^, including an overall increased bacterial diversity, an over-representation of *Prevotella copri, Dialister,* and *Eubacterium* and correspondingly depletion in *Bifidobacterium* and *Faecalibacterium* and *Clostridium*. Village C is characterized by lower education levels, lower monthly income, and considerably fewer Westernized exposures as compared to the higher SES villages. Although the traditional diet in Arab population integrates patterns of the traditional Mediterranean diet, in recent years consumption of processed carbohydrates has increased^43,44^. Correspondingly, the dietary patterns of Arab children in Israel are characterized by higher consumption of plant-based food, yet also an increased intake of savory sweets and soft drinks^45^. Albeit the growing exposure to processed carbohydrates in the Arab community, the diverging microbial signatures between the villages, and the over-representation of bacteria associated with rural communities in the lower SES village, might be affected by home cooking practices, the type and amounts of protein and the ability to purchase more processed food items.

Furthermore, there was a significant increase in the relative abundance of *Oscillospira* and *Sutterella* in association with lower SES. *Oscillospira* is considered an enigmatic bacterial genus that has never been cultured, however, was strongly associated with the production of butyrate, leanness and the utilization of mammalian-derived glycans, originating either from the host or from a diet rich in animal glycoproteins^46,47^. Similarly, although *Sutterella* is a commonly abundant genus in both children and adults, results concerning its association with human health and disease are controversial^48–51^. Given the strong evidence linking these genera to human health, further exploration is essential, given their association with low SES as demonstrated herein, and the pivotal role of social disparities in health and disease.

An analysis of the microbial differences in relation to household crowding revealed that *Alistipes onderdonkii*, *Bacteroides uniformis*, *Prevotella stercorea*, *Phascolarctobacterium,* and *Alistipes putredinis* were the taxa most strongly associated with household crowding, with a slightly lower detection level for *Faecalibacterium prausnitzii* and *Prevotella copri*. In the lower SES village, *Bacteroides uniformis* was mostly associated with household crowding, while the most dominant taxa in the higher SES villages were *Prevotella stercorea* and *Phascolarctobacterium*. The stratified analysis by residential village suggests that the disparities in SES were independently associated with the different relative abundance of *Alistipes putredinis*, *Eubacterium biforme*, *Dialister*, *Bifidobacterium*, *Oscillospira*, *Ruminococcus*, and *Sutterella*, regardless of the homogenous geographic location, ethnic, and dietary habits.

An inferred analysis of functional predictions identified three significantly differentially abundant metabolic pathways in association with village SES. D-Glutamine and D-glutamate metabolism, secondary bile acid biosynthesis and Biotin metabolism were depleted among participants from the lower SES than the higher SES villages. Children from the higher SES villages had an over-representation of secondary bile acid biosynthesis. Bile acid conversion is an important metabolic feature of the gut microbiota that affects the host metabolism, by regulation of metabolic and inflammatory signaling pathways^52^ and potent antimicrobial properties^53^.

Participants from the higher SES villages had increased glutamate and glutamine metabolism. Glutamine is a beneficial amino acid, which is utilized by the intestinal endothelium and plays a pivotal role in gut integrity^54,55^. Depleted glutamine levels were observed in inflammatory bowel disease^56^, obesity and adipose inflammation^57,58^, and gut-brain axis disorders^59^. Glutamate metabolized by gut microbiota was shown to be associated with human health, including decreased abundance in obesity^60^ insulin resistance^61^, and neurological disorders such as autism spectrum disorder^62^, seizures^63^, and Alzheimer’s disease^64^.

Biotin metabolism was greater in the higher SES villages than the low SES village. Biotin (vitamin B7) is an indispensable cofactor for several carboxylases important for glucose, amino acid, and fatty acid metabolism^65^. Biotin is available from food; however, several microbial species in the gut microbiome can synthesize biotin^66^. Biotin metabolism genes are found across different phyla suggesting that they have a core function in gut microbiome metabolism. Indeed, metagenomic analyses suggested an association between vitamin metabolism-related pathways and intestinal dysbiosis^67^, type 2 diabetes or inflammatory bowel disease^66,68^. Collectively these findings support the notion that the gut microbiome plays an important role in children’s health, likely through microbial and functional metabolic features that vary according to community SES and household crowding conditions. Our findings provide a basis for large scale, metagenomic and metabolomic longitudinal studies, to explore the clinical relevance of the observed metabolic differences according to SES.

Our study consisted of healthy children, which might explain the weak association between antibiotic use and the gut microbiome compared to SES and household crowding.

The current study has several strengths. First, this is a population-based study to explore the association between residential and household SES and the intestinal microbiome of school-age children, revealing that the microbiome is continuously influenced by environmental exposures in later childhood. This is specifically important since the childhood period is crucial for physical and cognitive development^69^. Second, data collection was performed using validated and standard tools, and the analysis included interpersonal covariates, using a model adjusted for age, sex, and household crowding index. Third, this study excluded the potential effect of ethnicity and geography on the microbial ecology, since all participants share these features. Although we did not include an analysis of dietary habits, the participants in this cohort share relatively similar cultural dietary patterns. Moreover, the villages included shared similar patterns of early life practices, including a near-universal breastfeeding and daycare attendance at a relatively late age (most participants enrolled on kindergartens at ages 3–5 years, the ages of pre-obligatory education in Israel). This in addition to the large sample size with SES gradient, minimizing the effects of possible microbiome determinants.

Our study has some limitations. First, this is a cross-sectional analysis that cannot provide answers on how SES affects the development and trajectories in the gut microbiome diversity and composition. However, SES and living conditions are pre-determined for children who depend on their parents, thus the directionality of the associations in our study is that SES affects the gut microbiome in childhood and not vice versa. These findings provide solid evidence and stimulate further longitudinal studies to explore the influence of SES disparities on the development and maturation of the gut microbiome. Second, we focused on SES factors, with the assumption that dietary habits are relatively alike in terms of cultural habits. Nevertheless, dietary habits might vary across the villages and households. While we revealed relative similarities in early life exposures, including a high prevalence of breastfeeding, late entrance to daycare, and the qualitative diet assessment showing similar consumption of main food groups (e.g., fruit, vegetables, meat, poultry) at ages 3–5 years, the lack of dietary intake information at age 6–9 years, is a limitation of our study. Thus, there is a need for an in-depth understanding of the development of these children’s nutrition prospectively during later childhood and the possible effect on the growing child’s microbiome. Third, in this analysis we used 16 S rRNA gene sequencing for inferences on microbial functional ecology. This broad-brush approach has limitations for drawing conclusions about changes in functional ecology. Nevertheless, our results add insight into the relationship between SES, intestinal microbiome and metabolic pathways in childhood, encouraging further examination in large-scale metagenomic studies. Finally, the inclusion of participants from a shared ethnic group and geographic location is a double-edged sword, since while minimizing the effect of these confounders, the role of SES disparities on the gut microbiome needs to be confirmed in other populations. Interestingly, even with focusing on a specific ethnic group, we observed significant associations with the “rural” microbiome, in line with existing studies^70^.

In conclusion, we demonstrated significant associations between SES disparities and intestinal microbiome differences in school-age healthy children. Importantly, our findings are in line with recent evidence^71,72^ showing that the microbiome continues to be strongly associated with environmental exposures through later childhood. These findings add new evidence to the emerging body of knowledge showing that both household and community levels characteristics are important determinants of the gut microbiome diversity and composition in healthy children, suggesting that the development of the gut microbiome might differ across and within populations. These continuous and persistent environmental exposures may be of great importance in the child’s development and health outcomes in adulthood. Since SES is associated with health disparities, further research is necessary to explore possible implications of SES-related microbiome differences on children’s health and development. A clearer understanding of the relationship between SES, the developing microbiome and the child’s health and wellbeing may possibly lead to more “population” or “neighborhood” personalized interventions for the promotion of health in more vulnerable communities.

## Methods

### Study population and design

The study included healthy Arab children aged 6 to 9 years, who participated in a study on enteric infections in Israel^23,73–77^. Briefly, in 2003–2004, a cohort of 289 healthy children aged 3–5 years from three neighboring Arab villages in Hadera sub-district in the northern region of Israel was followed for the incidence of diarrheal diseases and *H. pylori* infection prevalence^23,74^. In 2007–2009, a follow-up study was conducted at age 6–9 years, in which stool samples were obtained from 192 children. Inclusion criteria were birth at a gestational age of 34 weeks or more and birth weight of two kilograms (kg) or more^73,75–77^.

The Arab population is the main indigenous minority in Israel, comprising 20% of the population in Israel, while the Jewish population comprises 75% of the population and the rest belong to other ethnic groups^28,78^. It is in a positive transition with continuous improvement in infrastructure and educational levels. Despite this progress, the Arab population is of lower SES compared to the Jewish population^28,78^. All Israeli citizens have health insurance according to the national health insurance law implemented in Israel since 1995^79^.

During the study period, two of the study villages had ~12,000 residents each while the third village had ~17,000 residents. The villages were specifically selected to represent various socioeconomic conditions. According to the Central Bureau of Statistics during the study period the SES ranks of the villages ranged from 2 to 4 on a scale of 1–10; the highest rank is 10, i.e., a higher rank represents a better SES^80^. Village A belonged to cluster 4 SES rank, village B belonged to cluster 3 SES rank and village C belonged to cluster 2 SES rank. At the national levels these villages represent low-intermediate SES, however, within the Israeli Arab population the villages represent high SES (village A), intermediate SES village (village B), and low SES (village C)^80^. This classification is supported by the fact that at the national level 71 (93%) out of 76 Arab towns/villages had SES ranks of 1–4, while four towns/villages had 5 SES ranks and only one town in the Galilee had 6 SES rank^80^. Moreover, all Arab towns/villages in the study district had SES ranks between 2 and 4 during the study period. Knowing this a-priori, we purposefully selected the study villages to provide representativeness of different SES strata within the Arab population and region^23,73–77^.

The villages are connected to piped water and sanitation infrastructure, have connections to telephone, internet, and cable television networks. The villages have primary care clinics managed by the health maintenance originations, maternal and children health clinics of the Ministry of Health provide preventive services, including childhood immunizations. School-age immunizations and pediatric screening tests are also provided.

Israeli Arabs are historically agrarian, whose dietary habits were shaped by the traditionally self-produced foods (e.g., wheat, olives, legumes, vegetables, fruit). Although this population group is undergoing an urbanization process, still a high proportion of Arabs adhere to ethnic dietary traditions, including a high intake of the foods historically produced by the rural Arab population, characterized by Mediterranean elements^44^, including olive oil, wheat-based whole grains, such as burgul, legumes, and wild and cultivated non-starchy vegetables. Nowadays, the traditional Arab diet assimilates Westernized modifications (e.g., replacement of whole grains with refined grains, increased consumption of meat dishes/animal fat). This process is driven by the loss of the traditional agricultural lifestyle, rapid urbanization, access to subsidized refined grains and, growing exposure and mixing outside the Arab community^81^. A national survey of the dietary patterns of Israeli school-age children found that Arab children tend to consume more fruit and vegetables, but also more savory sweets, including soft drinks, containing high amounts of sugar compared to Jewish children^43,44^.

### Subject characteristics and household composition

Information on household and socioeconomic characteristics was obtained by personal interviews held with the mothers by trained Arabic‐speaking interviewers. The questionnaire included information on age, sex, place of residence, monthly family income (below, around, or above the national average), parental number of schooling years, number of siblings, number of persons living in the household, and number of rooms in the household (including living room, but not including kitchen). The household crowding index was calculated as the number of people living in a household divided by the number of rooms in the household; higher values represent higher household crowding living conditions. This variable was analyzed both as a continuous variable and as a categorical variable categorized by tertiles as follows: lowest tertile (0.6–1.25), middle tertile (1.26–2.0), and highest tertile (2.1–7.0) representing low, medium, and high household crowding, respectively. Information was also obtained on the child’s health status including antibiotic use during the six months prior to enrollment and diarrheal disease in the past year. Information was collected on early life exposures, such as breastfeeding, age of introducing solid foods, and attendance of daycare center. At age three to five years, a qualitative assessment of consumption of fruit, vegetables, poultry, and red meat was undertaken based on maternal reports via interviews. Mothers were asked whether the child regularly eats these items. The replies were yes or no.

At age six to nine years, measurements of the children’s weight (in kg) and standing height (in cm) were performed by the study-trained registered nurses. Bodyweight was measured to the nearest 0.1 kg using an analogue scale (calibrated before use), and height (to the nearest 0.1 cm) with a mobile stadiometer. BMI was calculated according to the formula (weight [kg]/ height [meter]^2^). BMI for age Z-score (BMIZ) was calculated using the 2000 reference population of the US Centers for Disease Control and Prevention (CDC)^82^ using Epi/Info software.

### Samples collection, DNA extraction, and bacterial DNA amplification

Mothers were instructed to collect stool samples from the participating child while the child was healthy and not during an acute illness. Stool samples were collected using collection plastic cups and transferred on ice to the study laboratory at Tel Aviv University. Samples were divided into two aliquots and stored at −80 °C until testing. All samples underwent a single thaw prior to DNA extraction.

Genomic DNA was extracted from 180 to 220 mg of fecal material from each sample using the QIAamp® Fast DNA Stool Mini Kit (Qiagen, Valencia, CA) according to the manufacturer’s instructions^83^ and stored at −20 °C until shipment to the Sequencing Core at the University of Illinois.

Genomic DNA was prepared for sequencing using a two-stage amplicon sequencing workflow^84^. Initially, genomic DNA was amplified via PCR using primers targeting the V4 region of microbial 16 S ribosomal RNA (rRNA) genes. The primers, 515 F modified and 806 R modified^85^, contained 5’ linker sequences compatible with Access Array primers for Illumina sequencers (Fluidigm, South San Francisco, CA). The PCR assays were performed in a total volume of 10 µL using MyTaq™ HS 2X Mix (Bioline) with primer concentrations at 500 nM. Thermocycling conditions were as follows: 95 °C for 5 min (initial denaturation), followed by 28 cycles of 95 °C for 30 sec, 55 °C for 45 sec, and 72 °C for 30 sec. One microliter of the PCR product from each reaction was transferred to the second-stage PCR assay. Each second-stage reaction was conducted in a final volume of 10 µL using MyTaq^TM^ HS 2X Mix, and each well contained a unique pair of Access Array primers containing Illumina sequencing adapters, single index sample-specific barcode, and linker sequences. Thermocycling conditions were as follows: 95 °C for 5 min (initial denaturation), followed by 8 cycles of 95 °C for 30 sec, 60 °C for 30 sec, and 72 °C for 30 sec. Libraries were pooled and purified using 0.6× concentration of AMPure XP beads to remove short fragments below 300 bp. Pooled libraries were loaded onto a MiniSeq sequencer (Illumina, San Diego, CA) with 15% phiX spike-in and paired-end 2 × 153 base sequencing reads.

Negative no-template controls were included at every step of the library preparation. Gel electrophoresis of first and second step PCR products, as well as the more sensitive PicoGreen DNA quantification assay, were employed to verify successful amplification in the study samples, and lack of amplification in the negative control samples.

### Statistical analyses

Quality control analysis of demultiplexed pair-end joined reads was performed using the native Deblur^86^ workflow, following the construction of a phylogenetic tree (mafft-fasttree) and taxonomy assignment with QIIME2^87^. The quality process with Deblur uses sequence error profiles to obtain putative error-free sequences, referred to as s-OTU. Taxonomic composition was assigned to the s-OTUs using a pre-trained Naive Bayes classifier, trained on the Greengenes^88^ 13_8 99% OTUs. Downstream analysis was conducted using R version 3.6.2. Diversity analysis was calculated at a rarefaction depth of 7479. Bacterial α-diversity was estimated using the number of observed s-OTUs, Pielou’s evenness, Shannon’s diversity and the Faith’s phylogenetic diversity (Faith’s PD) indexes and compared across independent variables using the Kruskal-Wallis test. β-diversity was calculated using the JSD and the phylogenetic Weighted Unifrac distances. PERMANOVA was used to test differences in overall microbiome composition (vegan; adonis^89^), implementing a multivariate model with the following covariates: age, sex, household crowding index, number of maternal schooling years, BMI (“full model”), and a “reduced model” that included age, sex, and household crowding index. Pairwise comparisons were calculated using Dunn test, and controlled for false discovery rate (FDR) with the Benjamini-Hochberg method (*p* < 0.05). Beta dispersion was estimated using “betadisper” function (vegan) and the Permutation test for homogeneity of multivariate dispersions was performed at 999 permutations.

The Analysis of Composition of Microbiomes^90^ (ANCOM) was applied for the identification of differentially abundant features across the study villages, with FDR level set to 0.05. ANCOM uses a linear framework to statistically detect features whose composition varies across the villages and household crowding index (analyzed as tertiles), while controlling for other covariates of interest (a linear model comprised of the abovementioned covariates). A feature was considered significantly varying in composition across an independent variable of interest at a detection level of ≥0.7, meaning that the feature composition varied across the independent variable with respect to 70% of reference features. Non-parametric Spearman’s correlation coefficient was used to evaluate the association between α-diversity indexes and household crowding index. An analysis of inferred metagenome functions was performed implementing PICRUSt2^91^ (q2-picrust2). Differentially abundant metabolic pathways were detected using the compositional ANCOM analysis at KEGG level 3.

Differences in demographic characteristics across the study villages were examined using one-way analysis of variance (ANOVA) for continuous variables and the chi-square test for categorical variables. Post-hoc pairwise comparisons were conducted using the Bonferroni test to correct for multiple comparisons.

#### Sensitivity analyses

The analyses were repeated while including in the models antibiotic use during the past 6 months and in relation to the history of diarrheal diseases in the past year prior to sampling collection and enrollment, as covariates.

Differences in microbial β-diversity according to the village of residence and household crowding were validated by implementing ANOSIM, a non-parametric test, in addition to the PERMANOVA models.

Since the sample size differed across the study villages (Results section) we examined whether the associations of residential SES and household crowding with the gut microbiome are affected by the sample size of the groups while utilizing a sub-sampling and re-analysis approach. We selected random and equal subsamples of the three villages (using dplyr package, R program), so each village included 47 randomly selected participants (the lowest sample size of village A) and reanalyzed the data using the subsamples.

### Ethical aspects

The Institution Review Board of Hillel Yaffe Medical Center and the ethics committee of Tel Aviv University approved the study. Written informed consent was obtained from the parents of the participants.

### Reporting summary

Further information on research design is available in the Nature Research Reporting Summary linked to this article.

## Supplementary information

Supplementary figures and tables

Reporting Summary Checklist

## Data Availability

All relevant data are available from the corresponding author: K.M. Data from this study cannot be publicly available due to legal and ethical restrictions.
