# Supplementary material for: Socioeconomic disparities and household crowding in association with the fecal microbiome of school-age children
Source: NPJ Biofilms Microbiomes. 2022 Mar 3;8:10. doi: 10.1038/s41522-022-00271-6 (PMC8894399; doi:10.1038/s41522-022-00271-6)
Supplement: Supplementary file 1 — Supplementary figures and tables [file 41522_2022_271_MOESM1_ESM.pdf]

Supplementary figure 1: Household crowding, mother's education and village of residence

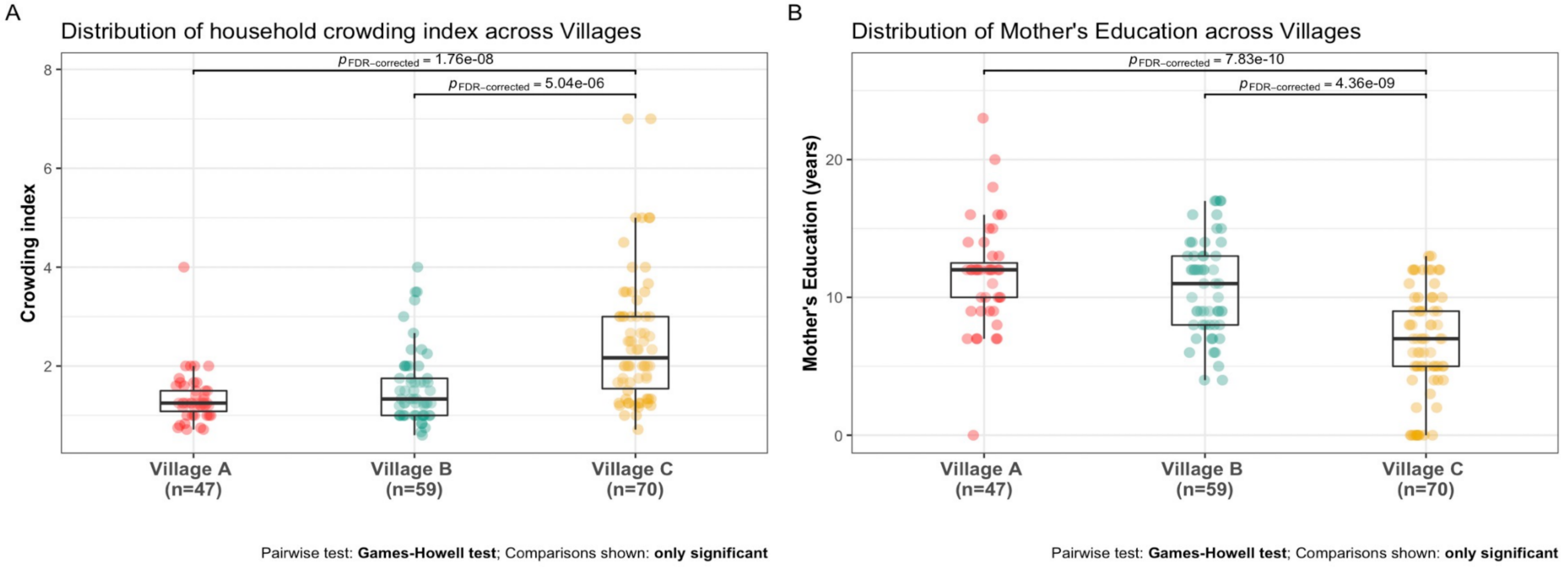

(A) Boxplots of the household crowding index by village. The mean crowding index was significantly higher in village C compared to village B (p<0.001) and village A (p<0.001), but not between villages A and B (p=0.212).

(B) Boxplots of the distribution of mother's education level (years) by village. The mean mother's education was significantly lower in village C compared to village B (p<0.001) and village A (p<0.001), but not between villages A and B (p=0.363). The sample size in each village was 47, 59 and 70, respectively.

Supplementary figure 2: Pie charts of early life dietary intake reported by the participants' mothers showing similar exposures in villages A and B compared to village C.

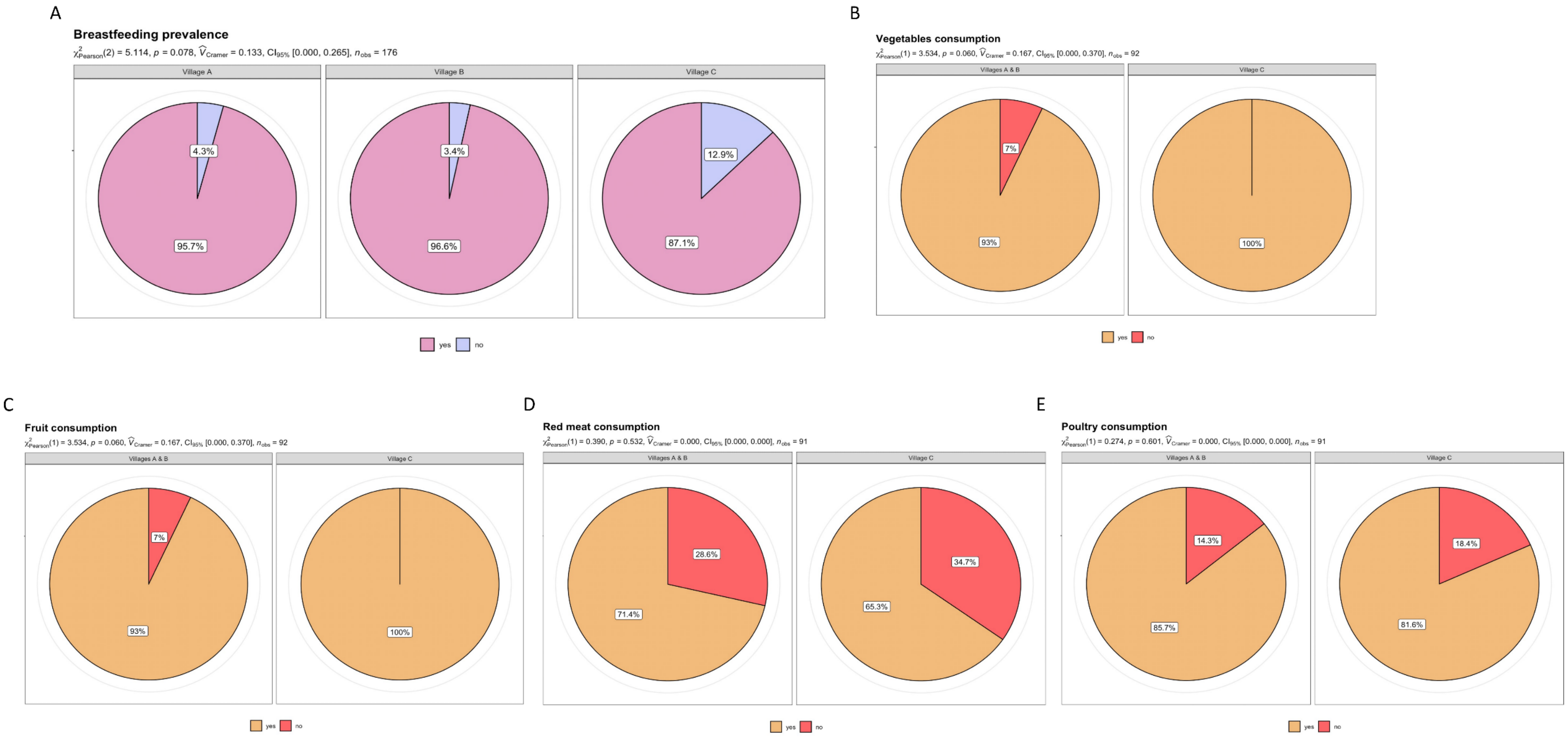

There were no significant divergences in **(A)** breastfeeding prevalence (p=0.101), **(B-C)** consumption of vegetables and fruit (p=0.06 for both comparisons), **(D-E)** and intake of red meat and poultry (p=0.532 and p=0.601, respectively).
The sample size villages A and B was 44, and 49 in village

Supplementary figure 3: Household crowding, mother's education and village of residence in sub-samples

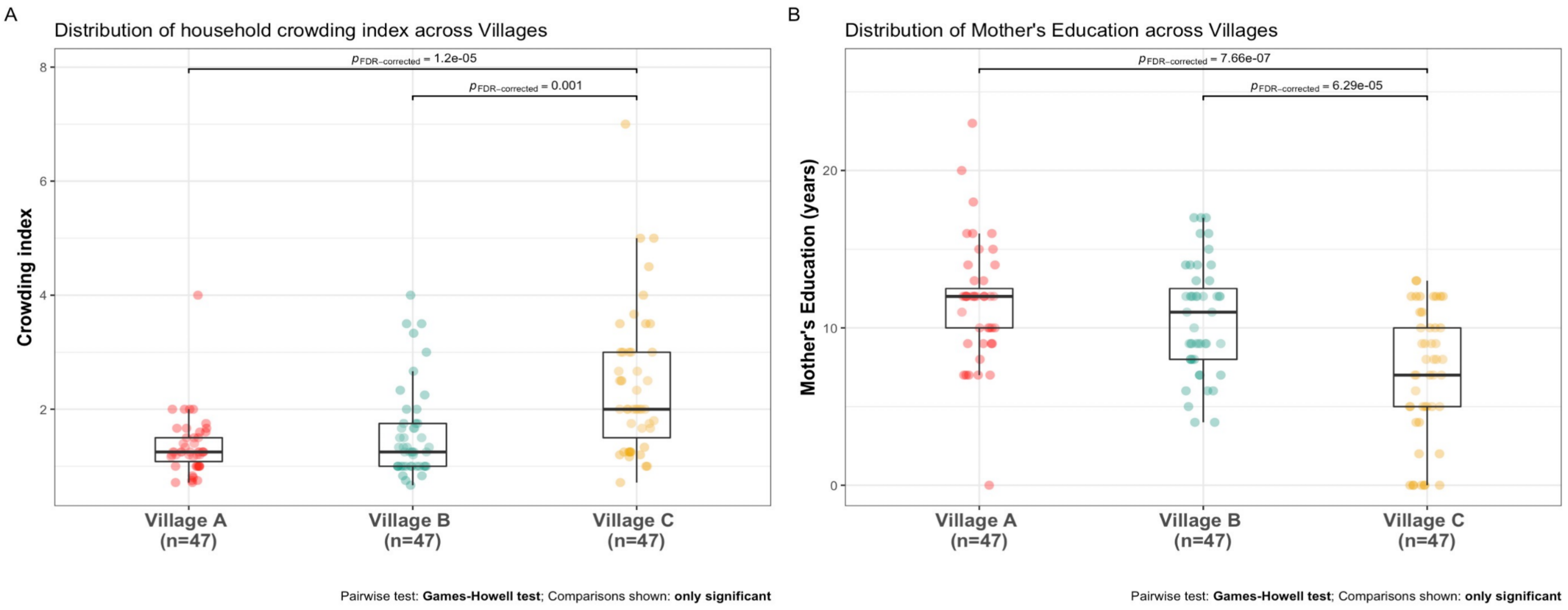

(A) Boxplots of the household crowding index across the random subsamples of the three villages (n=47 in each village). The mean crowding index was significantly higher in village C compared to village B ($p=0.001$) and village A ($p<0.001$), but not between villages A and B ($p=0.265$). **(B)** Boxplots of the distribution of mother's education level (years) across in the random subsamples of the three villages. The mean mother's education was significantly lower in village C compared to village B ($p<0.001$) and village A ($p<0.001$), but not between villages A and B ($p=0.298$).

Supplementary figure 4: α-diversity in the subsampled groups by village.

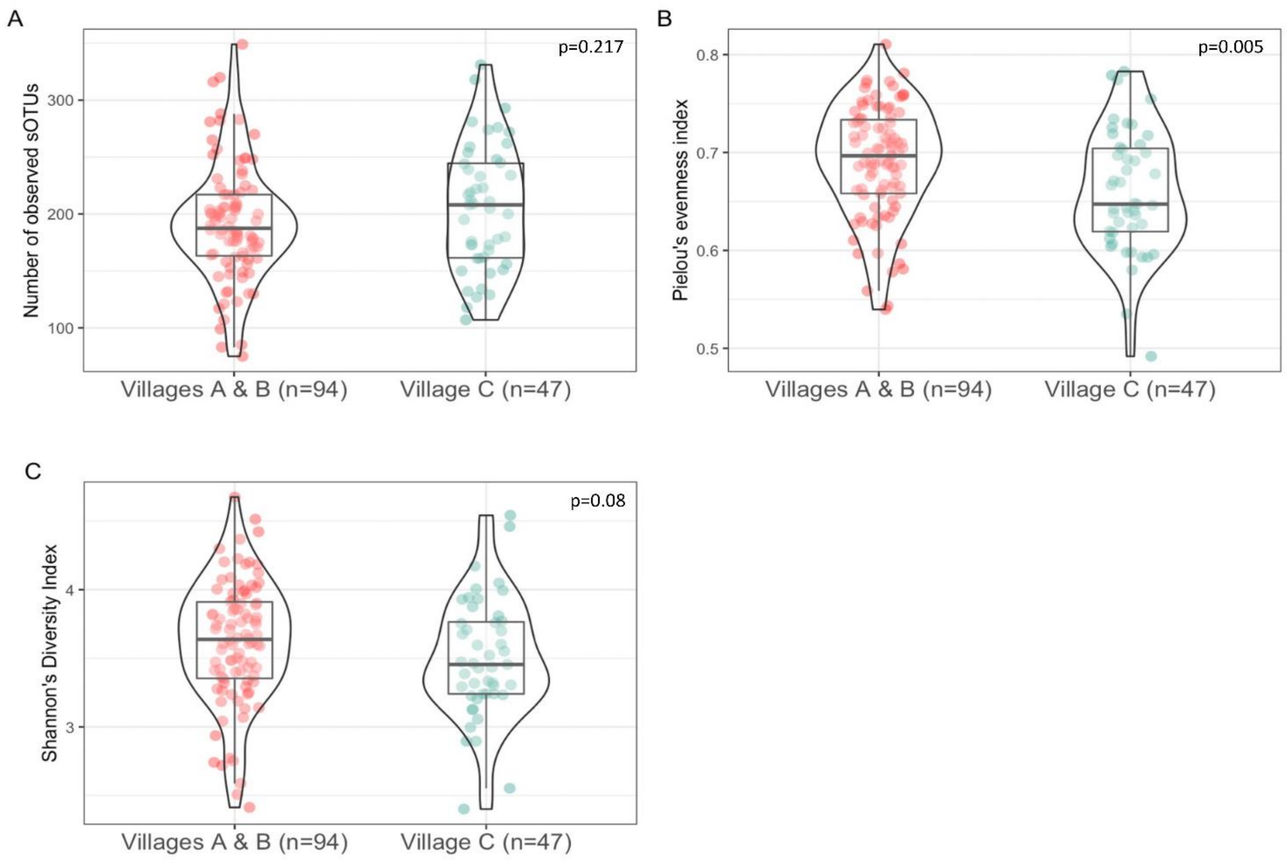

(A) Box-violin plots of microbial richness, measured by the number of observed s-OTUs
(B) Box-violin plots of microbial α-diversity, measured by Pielou’s evenness index.
(C) Box-violin plots of microbial α-diversity, measured by the Shannon’s diversity index. P value for the differences between villages A and B vs. village C: p=0.217 for the number of observed s-OTUs, p=0.005 for Pielou's evenness index, p=0.08 for Shannon’s diversity. The sample size was 47 in each village.

Supplementary figure 5: Microbiome composition in the subsampled groups by village

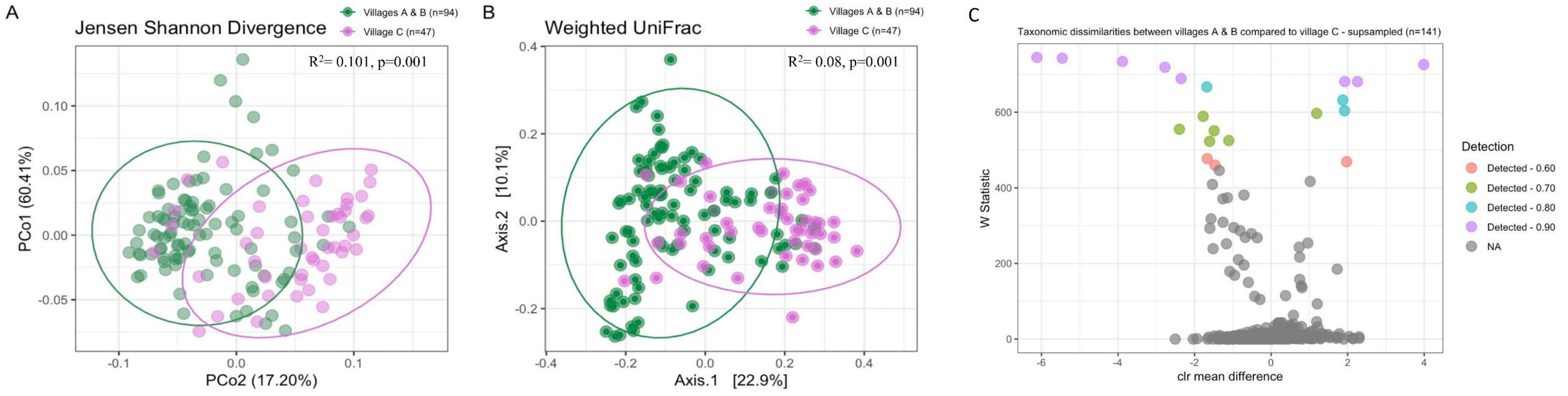

(A) Principal coordinate analysis of the JSD notably different among children villages A and B vs. village C (PERMANOVA R^2= 0.101, p=0.001). **(B)** Principal coordinate analysis of the weighted UniFrac notably different among children villages A and B vs. village C (PERMANOVA R^2= 0.08, p=0.001). **(C)** Volcano plot showing differentially abundant s-OTUs as detected by ANCOM, between the subsampled villages A and B compared to village C. The *x*-axis represents the difference in mean centered log ratio (clr)-transformed abundance between groups and the *y*-axis represents the ANCOM W Statistic. s-OTU points are colored by level of ANCOM significance, with 0.9 being the highest level; s-OTUs in gray were not significant. The sample size was 47 in each village.

Supplementary figure 6: Household crowding and α-diversity in the subsampled groups

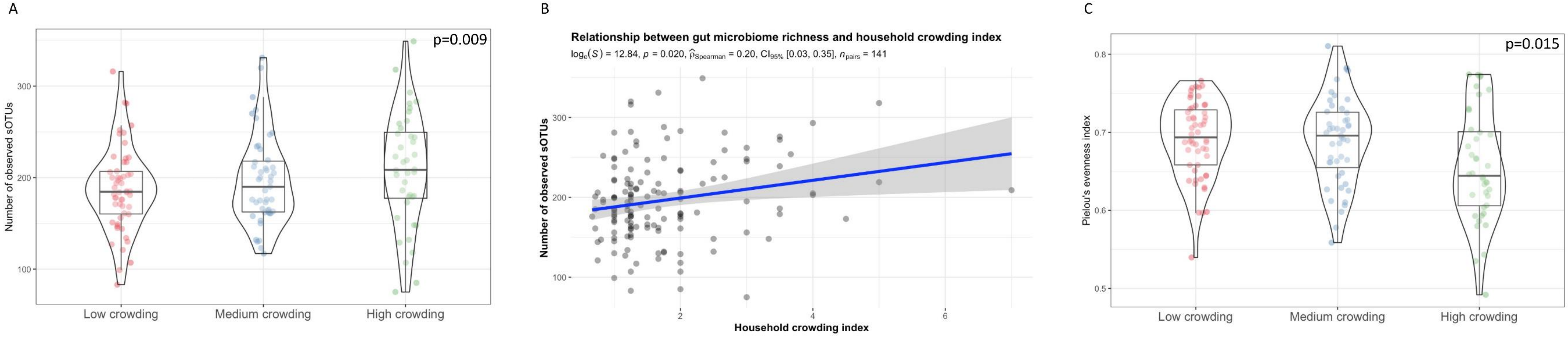

(A) Box-violin plots of microbial richness, measured by the number of observed s-OTUs increased with higher household crowding (p=0.009).
(B) Spearman's correlation between microbial richness and household crowding (p=0.02)
(C) Box-violin plots of microbial α-diversity, measured by Pielou's evenness index, decreased with higher household crowding (p=0.015). The sample size was 47 in each village.

Supplementary figure 7: Household crowding and microbiome composition in the subsampled groups.

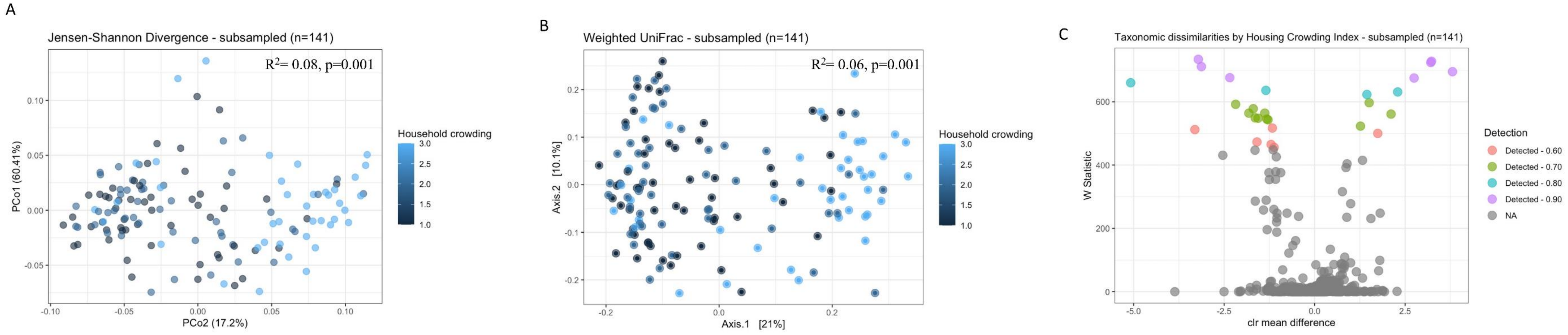

(A) Principal coordinate analysis of the JSD notably different with differing household crowding (PERMANOVA R^2=0.08, p=0.001).
(B) Principal coordinate analysis of the weighted UniFrac notably different across differing household crowding (PERMANOVA R^2=0.06, p=0.001).
(C) Volcano plot showing differentially abundant s-OTUs as detected by ANCOM, with varying household crowding in the subsampled cohort (n=141). The *x*-axis represents the difference in mean centered log ratio (clr)-transformed abundance between groups and the *y*-axis represents the ANCOM W Statistic. s-OTU points are colored by level of ANCOM significance, with 0.9 being the highest level; s-OTUs in gray were not significant. The sample size was 47 in each village.

Supplementary figure 8: Volcano plot showing differentially abundant s-OTUs and clr-transformed abundance of s-OTUs – villages A-B

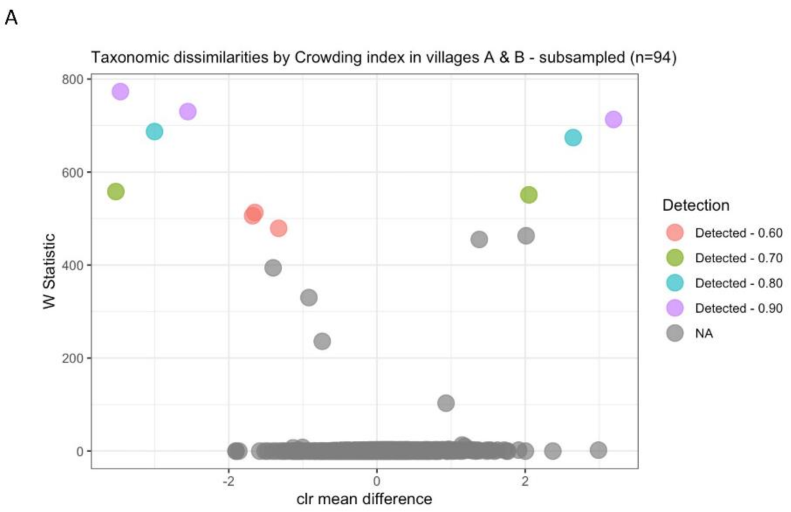

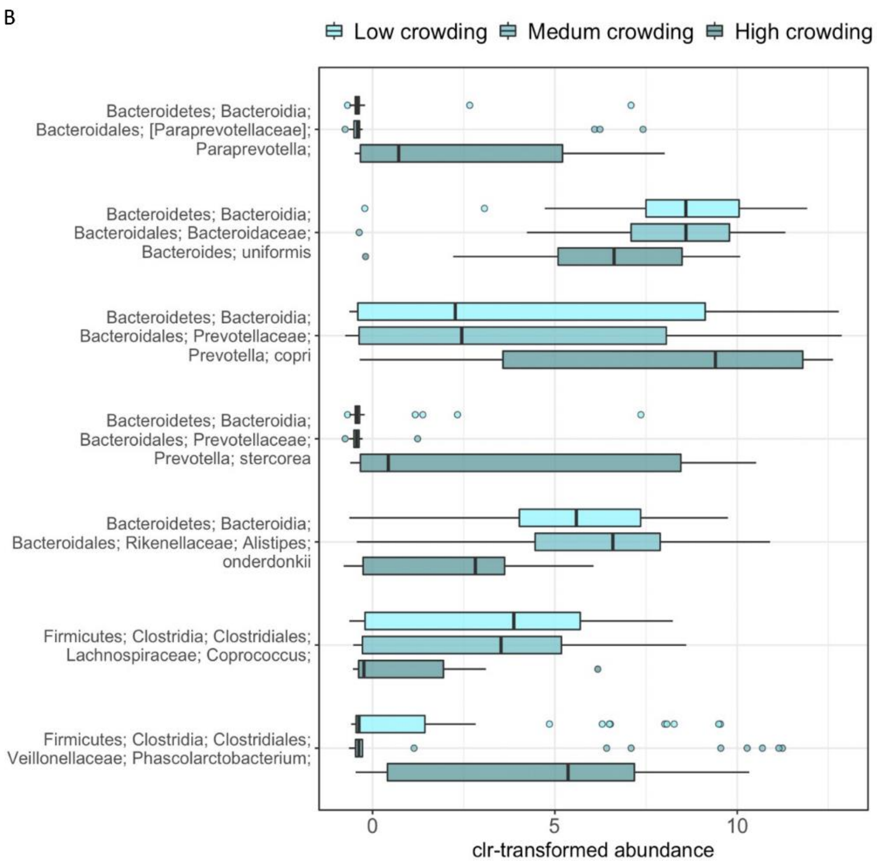

(**A**) Volcano plot showing differentially abundant s-OTUs as detected by ANCOM, with varying household crowding in the subsampled villages A and B (n=93). The *x*-axis represents the difference in mean centered log ratio (clr)-transformed abundance between groups and the *y*-axis represents the ANCOM W Statistic. s-OTU points are colored by level of ANCOM significance, with 0.9 being the highest level; s-OTUs in gray were not significant. (**B**) Boxplots of clr-transformed abundance of s-OTUs significantly associated with the household crowding index in villages A and B [higher SES], adjusted for sex, age and village of residence. Tertiles of crowding index were categorized as low, middle and high household crowding tertiles.

Supplementary figure 9: Volcano plot showing differentially abundant s-OTUs and clr-transformed abundance of s-OTUs – village C

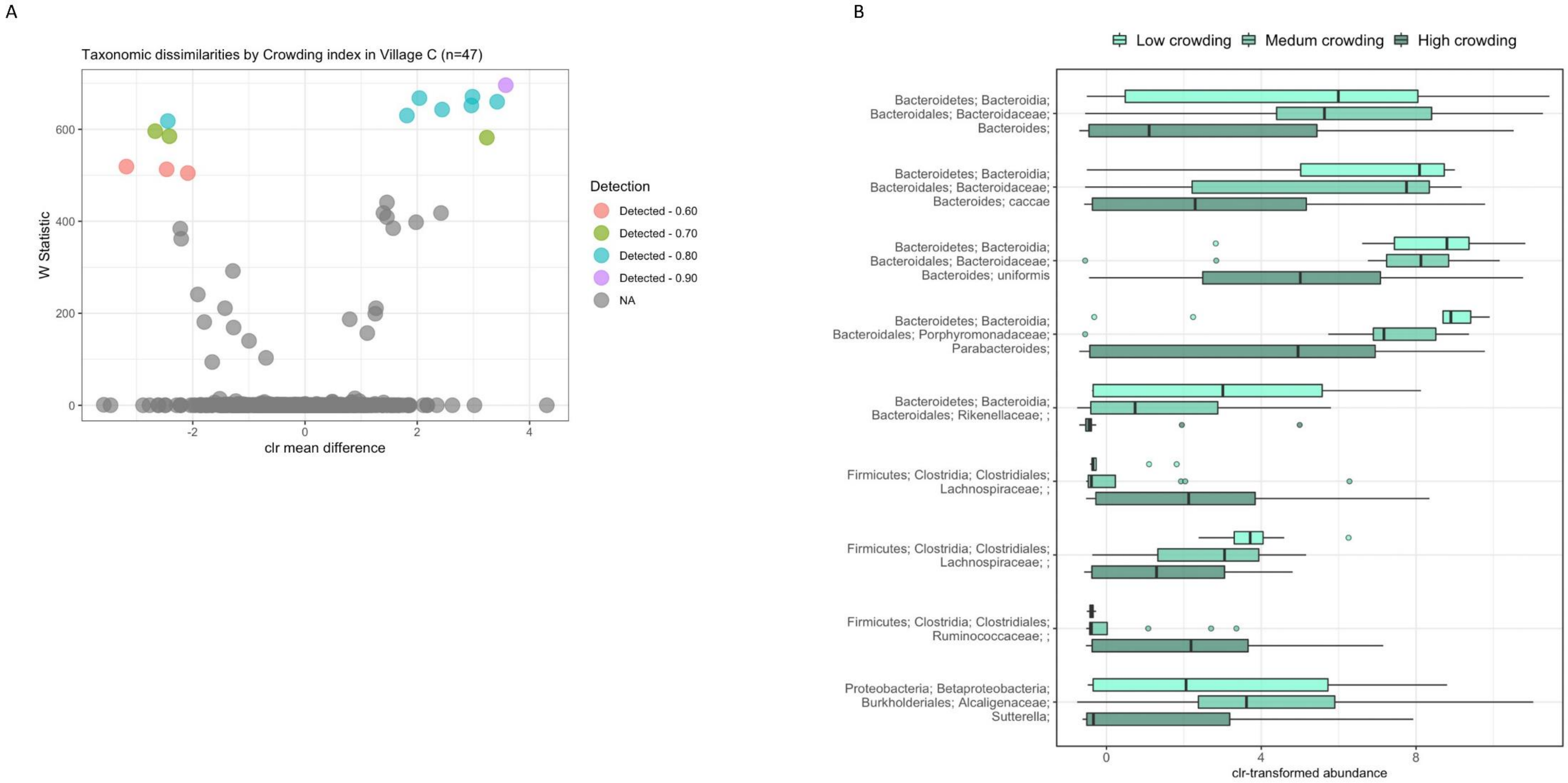

(**A**) Volcano plot showing differentially abundant s-OTUs as detected by ANCOM, with varying household crowding in the subsampled village C (n=47). The *x*-axis represents the difference in mean centered log ratio (clr)-transformed abundance between groups and the *y*-axis represents the ANCOM W Statistic. s-OTU points are colored by level of ANCOM significance, with 0.9 being the highest level; s-OTUs in gray were not significant. (**B**) Boxplots of clr-transformed abundance of s-OTUs significantly associated with the household crowding index in village C [lower SES], adjusted for sex, age and village of residence. Tertiles of crowding index were categorized as low, middle and high household crowding tertiles.

Supplementary figure 10: The gut microbiome and antibiotics use

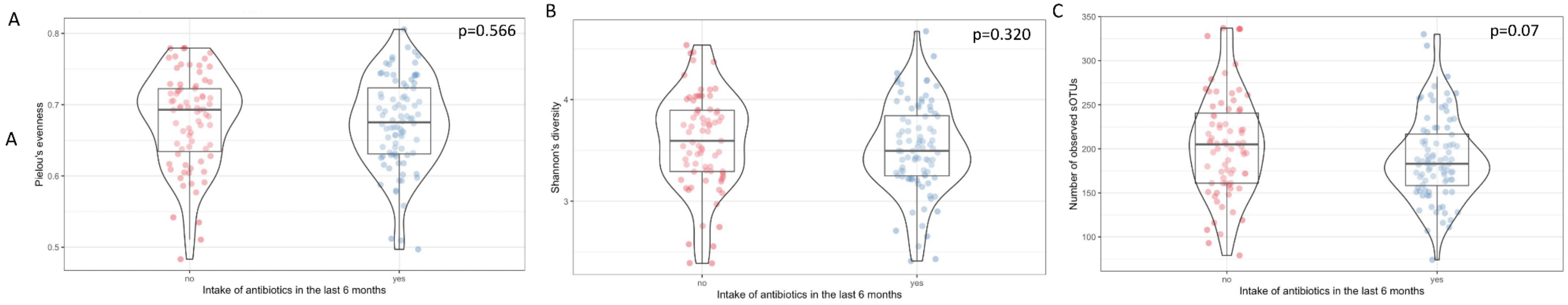

D

Weighted UniFrac	Df	R2	F	Pr(>F)
village	1	0.087	15.530	0.001
age	1	0.007	1.340	0.142
sex	1	0.009	1.580	0.059
crowding index	1	0.016	2.870	0.002
mother's education (y)	1	0.004	0.760	0.738
BMI	1	0.004	0.658	0.877
Antibiotics (last 6 months)	1	0.008	1.354	0.148
Residual	155	0.865	NA	NA
Total	162	1.000	NA	NA

E

Jensen Shannon Divergence	Df	R2	F	Pr(>F)
village	1	0.119	22.297	0.001
age	1	0.008	1.510	0.115
sex	1	0.009	1.628	0.084
crowding index	1	0.020	3.777	0.002
mother's education (y)	1	0.004	0.809	0.614
BMI	1	0.004	0.803	0.595
Antibiotics (last 6 months)	1	0.011	2.135	0.031
Residual	155	0.825	NA	NA
Total	162	1.000	NA	NA

F

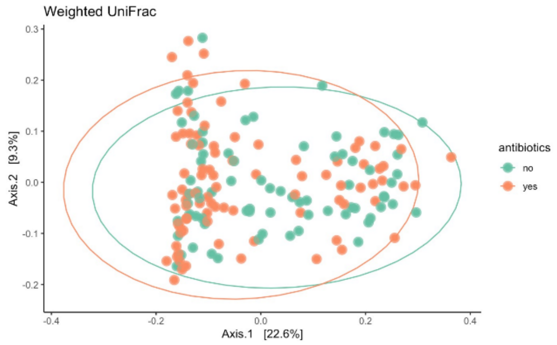

G

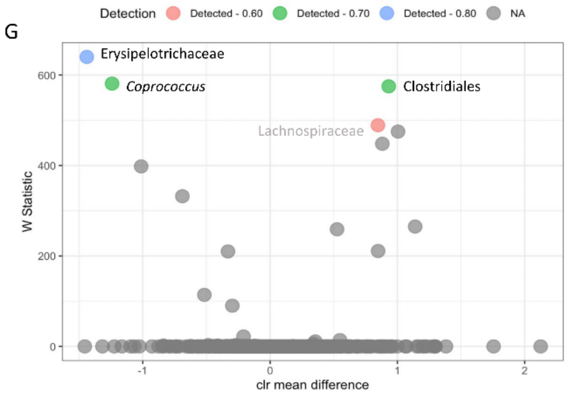

(A) Box-violin plots of microbial richness, measured by the Pielou's evenness index. **(B)** Box-violin plots of microbial α-diversity, measured by Shannon's diversity index. **(C)** Box-violin plots of microbial α-diversity, measured by the number of observed s-OTUs. P value for the differences between children who received antibiotics in the last six months compared to those who did not: p=0.566 for Pielou's evenness index, p=0.320 for Shannon's diversity, and p=0.07 for the number of observed s-OTUs**.** **(D-E)** Multivariate PERMANOVA models of the weighted UniFrac and the JSD, including antibiotics use as a covariate. **(F)** Principal coordinate analysis of the weighted UniFrac showing no significant separation between children that received antibiotics compared to their counterparts. **(G)** Volcano plot showing differentially abundant s-OTUs as detected by ANCOM. The *x*-axis represents the difference in mean centered log ratio (clr)-transformed abundance between groups and the *y*-axis represents the ANCOM W Statistic. s-OTU points are colored by level of ANCOM significance, with 0.8 being the highest level; s-OTUs in gray were not significant.

Supplementary figure 11: Gut microbiome and history of diarrheal episodes

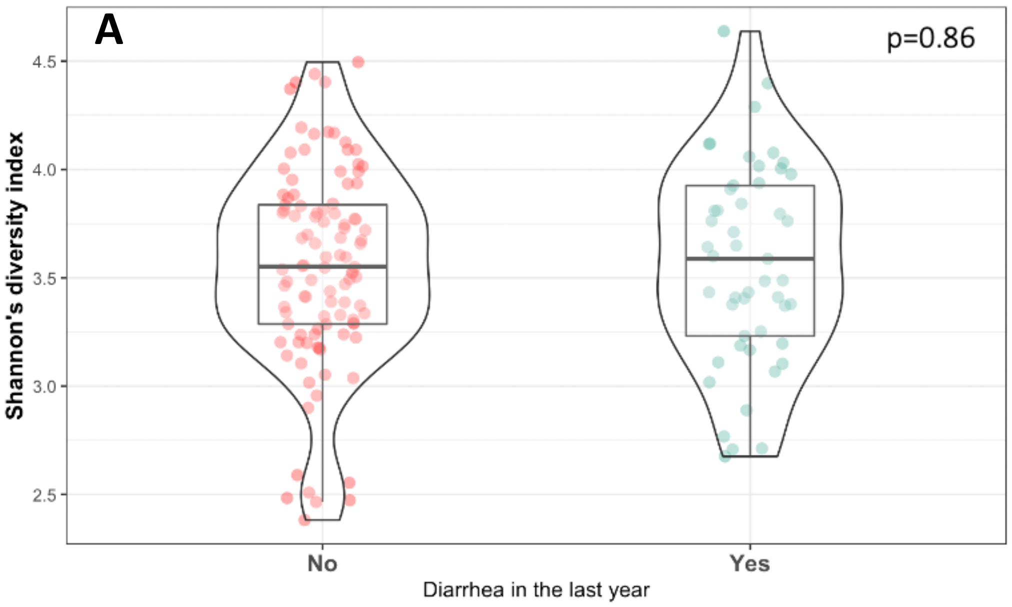

B: Jensen Shannon Divergence					
	Df	SumOfSqs	R2	F	Pr(>F)
Village	1	0.659	0.034	6.896	0.001
Age	1	0.133	0.007	1.387	0.14
Sex	1	0.140	0.007	1.465	0.103
Household crowding	1	0.311	0.016	3.251	0.003
Mother's education (years)	1	0.078	0.004	0.811	0.623
BMI	1	0.106	0.006	1.108	0.282
Diarrhea (last year)	2	0.163	0.009	0.854	0.667
Residual	167	15.971	0.835	NA	NA
Total	175	19.136	1.000	NA	NA

C: Weighted UniFrac					
	Df	SumOfSqs	R2	F	Pr(>F)
Village	1	0.638	0.029	5.594	0.001
Age	1	0.164	0.007	1.438	0.110
Sex	1	0.157	0.007	1.381	0.125
Household crowding	1	0.289	0.013	2.537	0.005
Mother's education (years)	1	0.076	0.003	0.665	0.855
BMI	1	0.119	0.005	1.043	0.357
Diarrhea (last year)	2	0.167	0.008	0.732	0.896
Residual	167	19.038	0.862	NA	NA
Total	175	22.081	1.000	NA	NA

(**A**) Box-violin plots of microbial richness, measured by the Shannon's diversity index. P value for the differences between children who had diarrheal episodes in the last year compared to those who did not: p=0.86.

(**B-C**) Multivariate PERMANOVA models of the weighted UniFrac and the JSD, including the incidence of diarrheal episodes in the last year

Supplementary table 1: PERMANOVA results- reduced model (n=176)						
(A) Jensen Shannon Divergence						
	Df	SumsOfSqs	MeanSqs	F.Model	R2	Pr(>F)
Village	1	2.176	2.176	22.792	0.114	0.001
Age, years	1	0.150	0.150	1.570	0.008	0.076
Sex	1	0.153	0.153	1.607	0.008	0.076
Crowding index	1	0.350	0.350	3.668	0.018	0.002
Residuals	171	16.323	0.095	NA	0.852	NA
Total	175	19.152	NA	NA	1.000	NA
(B) Weighted UniFrac						
	Df	SumsOfSqs	MeanSqs	F.Model	R2	Pr(>F)
Village	1	2.091	1.046	10.511	0.107	0.001
Age, years	1	0.081	0.081	0.818	0.004	0.589
sex	1	0.167	0.167	1.677	0.009	0.066
Crowding index	1	0.290	0.290	2.912	0.015	0.004
Residuals	170	16.912	0.099	NA	0.865	NA
Total	175	19.542	NA	NA	1.000	NA
DF: degrees of freedom; NA: not applicable; PERMANOVA: Permutational multivariate analysis of variance						

Supplementary table 2: PERMANOVA results- full model (n=176)						
(A) Jensen Shannon Divergence						
	Df	SumsOfSqs	MeanSqs	F.Model	R2	Pr(>F)
Village	1	2.164	2.164	22.662	0.113	0.001
Age, years	1	0.153	0.153	1.597	0.008	0.07
Sex	1	0.149	0.149	1.563	0.008	0.094
Crowding index	1	0.351	0.351	3.672	0.018	0.004
Mother's education, (years)	1	0.079	0.079	0.828	0.004	0.608
BMI Z score	1	0.108	0.108	1.131	0.006	0.269
Residuals	169	16.140	0.096	NA	0.843	NA
Total	175	19.144	NA	NA	1	NA
(B) Weighted UniFrac						
	Df	SumsOfSqs	MeanSqs	F.Model	R2	Pr(>F)
Village	1	2.014	1.007	10.106	0.104	0.001
Age, years	1	0.087	0.087	0.872	0.004	0.53
Sex	1	0.117	0.117	1.178	0.006	0.221
Crowding index	1	0.290	0.290	2.910	0.015	0.006
Mother's education, (years)	1	0.084	0.084	0.840	0.004	0.606
BMI Z score	1	0.108	0.108	1.079	0.006	0.328
Residuals	168	16.740	0.100	NA	0.861	NA
Total	175	19.439	NA	NA	1	NA

BMI: Body mass index; DF: degrees of freedom; NA: not applicable; PERMANOVA: Permutational multivariate analysis of variance

Supplementary table 3 - ANCOM results adjusted for age, sex and household crowding index		
W_stat	Taxonomy	Detection Level
776	Bacteroidetes; Bacteroidia; Bacteroidales; Prevotellaceae; Prevotella; copri	0.9
767	Firmicutes; Clostridia; Clostridiales; Veillonellaceae; Dialister;	0.9
765	Bacteroidetes; Bacteroidia; Bacteroidales; Rikenellaceae; Alistipes; putredinis	0.9
762	Firmicutes; Erysipelotrichi; Erysipelotrichales; Erysipelotrichaceae; [Eubacterium]; biforme	0.9
747	Actinobacteria; Actinobacteria; Bifidobacteriales; Bifidobacteriaceae; Bifidobacterium;	0.9
740	Firmicutes; Clostridia; Clostridiales; Ruminococcaceae; Faecalibacterium; prausnitzii	0.9
723	Firmicutes; Clostridia; Clostridiales; Ruminococcaceae; Oscillospira;	0.9
721	Proteobacteria; Betaproteobacteria; Burkholderiales; Alcaligenaceae; Sutterella;	0.9
714	Firmicutes; Clostridia; Clostridiales; Clostridiaceae; Clostridium;	0.9
712	Firmicutes; Clostridia; Clostridiales; Ruminococcaceae; NA; NA	0.9
709	Bacteroidetes; Bacteroidia; Bacteroidales; Rikenellaceae; Alistipes; onderdonkii	0.9
708	Firmicutes; Clostridia; Clostridiales; Ruminococcaceae; Ruminococcus;	0.9
696	Bacteroidetes; Bacteroidia; Bacteroidales; Porphyromonadaceae; Parabacteroides; distasonis	0.8
694	Bacteroidetes; Bacteroidia; Bacteroidales; Bacteroidaceae; Bacteroides; ovatus	0.8
688	Firmicutes; Clostridia; Clostridiales; [Mogibacteriaceae]; ;	0.8
669	Firmicutes; Clostridia; Clostridiales; Veillonellaceae; Dialister;	0.8
661	Bacteroidetes; Bacteroidia; Bacteroidales; Prevotellaceae; Prevotella;	0.8
657	Bacteroidetes; Bacteroidia; Bacteroidales; Prevotellaceae; Prevotella;	0.8
656	Bacteroidetes; Bacteroidia; Bacteroidales; Bacteroidaceae; Bacteroides; uniformis	0.8
648	Firmicutes; Erysipelotrichi; Erysipelotrichales; Erysipelotrichaceae; Catenibacterium;	0.8
647	Firmicutes; Clostridia; Clostridiales; [Mogibacteriaceae]; ;	0.8
638	Firmicutes; Clostridia; Clostridiales; Ruminococcaceae; ;	0.8
633	Firmicutes; Clostridia; Clostridiales; Ruminococcaceae; Ruminococcus;	0.8
632	Bacteroidetes; Bacteroidia; Bacteroidales; Prevotellaceae; Prevotella;	0.8
627	Bacteroidetes; Bacteroidia; Bacteroidales; Prevotellaceae; Prevotella; stercorea	0.8
627	Firmicutes; Clostridia; Clostridiales; Ruminococcaceae; Oscillospira;	0.8
623	Bacteroidetes; Bacteroidia; Bacteroidales; Rikenellaceae; ;	0.8
615	Firmicutes; Clostridia; Clostridiales; ; ;	0.7
614	Firmicutes; Clostridia; Clostridiales; Ruminococcaceae; NA; NA	0.7
604	Bacteroidetes; Bacteroidia; Bacteroidales; Rikenellaceae; ;	0.7
602	Firmicutes; Clostridia; Clostridiales; Ruminococcaceae; Ruminococcus;	0.7
601	Firmicutes; Clostridia; Clostridiales; Ruminococcaceae; NA; NA	0.7
594	Bacteroidetes; Bacteroidia; Bacteroidales; [Barnesiellaceae]; ;	0.7
591	Firmicutes; Clostridia; Clostridiales; Ruminococcaceae; Ruminococcus;	0.7
588	Firmicutes; Clostridia; Clostridiales; Ruminococcaceae; NA; NA	0.7
588	Firmicutes; Clostridia; Clostridiales; ; ;	0.7
575	Bacteroidetes; Bacteroidia; Bacteroidales; Bacteroidaceae; Bacteroides;	0.7
571	Bacteroidetes; Bacteroidia; Bacteroidales; Bacteroidaceae; Bacteroides;	0.7
571	Bacteroidetes; Bacteroidia; Bacteroidales; Rikenellaceae; ;	0.7
571	Firmicutes; Clostridia; Clostridiales; Lachnospiraceae; ;	0.7
571	Firmicutes; Clostridia; Clostridiales; [Mogibacteriaceae]; ;	0.7
563	Firmicutes; Clostridia; Clostridiales; Ruminococcaceae; NA; NA	0.7
554	Tenericutes; Mollicutes; RF39; ; ;	0.7
553	Firmicutes; Erysipelotrichi; Erysipelotrichales; Erysipelotrichaceae; Catenibacterium;	0.7
552	Firmicutes; Clostridia; Clostridiales; Lachnospiraceae; ;	0.7
549	Firmicutes; Clostridia; Clostridiales; Christensenellaceae; ;	0.7
543	Proteobacteria; Betaproteobacteria; Burkholderiales; Alcaligenaceae; Sutterella;	0.6
541	Firmicutes; Clostridia; Clostridiales; Ruminococcaceae; Ruminococcus;	0.6
537	Firmicutes; Clostridia; Clostridiales; Ruminococcaceae; Ruminococcus; flavefaciens	0.6
537	Firmicutes; Clostridia; Clostridiales; Ruminococcaceae; Oscillospira;	0.6
535	Firmicutes; Clostridia; Clostridiales; Lachnospiraceae; Coprococcus;	0.6
534	Firmicutes; Clostridia; Clostridiales; Lachnospiraceae; Coprococcus; eutactus	0.6
526	Bacteroidetes; Bacteroidia; Bacteroidales; Bacteroidaceae; Bacteroides; NA	0.6
522	Firmicutes; Clostridia; Clostridiales; Christensenellaceae; ;	0.6
515	Firmicutes; Clostridia; Clostridiales; Ruminococcaceae; ;	0.6
514	Firmicutes; Clostridia; Clostridiales; Ruminococcaceae; ;	0.6
504	Bacteroidetes; Bacteroidia; Bacteroidales; Bacteroidaceae; Bacteroides;	0.6
494	Firmicutes; Clostridia; Clostridiales; Lachnospiraceae; NA; NA	0.6
493	Firmicutes; Clostridia; Clostridiales; ; ;	0.6
482	Firmicutes; Clostridia; Clostridiales; Ruminococcaceae; Ruminococcus;	0.6
478	Firmicutes; Clostridia; Clostridiales; Lachnospiraceae; Coprococcus;	0.6
477	Firmicutes; Clostridia; Clostridiales; ; ;	0.6
477	Firmicutes; Clostridia; Clostridiales; Ruminococcaceae; Oscillospira;	0.6
472	Firmicutes; Clostridia; Clostridiales; Ruminococcaceae; ;	0.6

ANCOM: Analysis of Composition of Microbiomes; BMI: body mass index (n=176)

Supplementary table 4 - ANCOM results adjusted for age, sex, household crowding index, BMI and mother's education level (n=176)		
W_stat	Taxonomy	Detection Level
774	Bacteroidetes; Bacteroidia; Bacteroidales; Prevotellaceae; Prevotella; copri	0.9
774	Bacteroidetes; Bacteroidia; Bacteroidales; Prevotellaceae; Prevotella; copri	0.9
769	Bacteroidetes; Bacteroidia; Bacteroidales; Rikenellaceae; Alistipes; putredinis	0.9
763	Firmicutes; Erysipelotrichi; Erysipelotrichales; Erysipelotrichaceae; [Eubacterium]; biforme	0.9
760	Firmicutes; Clostridia; Clostridiales; Veillonellaceae; Dialister;	0.9
733	Firmicutes; Clostridia; Clostridiales; Ruminococcaceae; Faecalibacterium; prausnitzii	0.9
716	Firmicutes; Erysipelotrichi; Erysipelotrichales; Erysipelotrichaceae; [Eubacterium]; biforme	0.9
709	Actinobacteria; Actinobacteria; Bifidobacteriales; Bifidobacteriaceae; Bifidobacterium;	0.9
704	Firmicutes; Clostridia; Clostridiales; Ruminococcaceae; Oscillospira;	0.9
703	Firmicutes; Clostridia; Clostridiales; Ruminococcaceae; Ruminococcus;	0.9
676	Proteobacteria; Betaproteobacteria; Burkholderiales; Alcaligenaceae; Sutterella;	0.8
673	Firmicutes; Clostridia; Clostridiales; Ruminococcaceae; Ruminococcus; flavefaciens	0.8
669	Proteobacteria; Betaproteobacteria; Burkholderiales; Alcaligenaceae; Sutterella;	0.8
668	Firmicutes; Clostridia; Clostridiales; Lachnospiraceae; Coprococcus;	0.8
666	Firmicutes; Clostridia; Clostridiales; Ruminococcaceae; NA; NA	0.8
653	Bacteroidetes; Bacteroidia; Bacteroidales; Rikenellaceae; Alistipes; onderdonkii	0.8
629	Firmicutes; Clostridia; Clostridiales; [Mogibacteriaceae]; ;	0.8
628	Bacteroidetes; Bacteroidia; Bacteroidales; Bacteroidaceae; Bacteroides; ovatus	0.8
611	Proteobacteria; Betaproteobacteria; Burkholderiales; Alcaligenaceae; Sutterella;	0.7
591	Firmicutes; Clostridia; Clostridiales; ; ;	0.7
573	Bacteroidetes; Bacteroidia; Bacteroidales; Bacteroidaceae; Bacteroides;	0.7
573	Firmicutes; Clostridia; Clostridiales; Ruminococcaceae; Oscillospira;	0.7
570	Firmicutes; Clostridia; Clostridiales; Ruminococcaceae; Oscillospira;	0.7
563	Bacteroidetes; Bacteroidia; Bacteroidales; Prevotellaceae; Prevotella;	0.7
563	Firmicutes; Clostridia; Clostridiales; [Mogibacteriaceae]; ;	0.7
562	Bacteroidetes; Bacteroidia; Bacteroidales; Porphyromonadaceae; Parabacteroides; distasonis	0.7
555	Bacteroidetes; Bacteroidia; Bacteroidales; Prevotellaceae; Prevotella;	0.7
546	Firmicutes; Clostridia; Clostridiales; Ruminococcaceae; NA; NA	0.7
545	Firmicutes; Clostridia; Clostridiales; Ruminococcaceae; Ruminococcus;	0.7
536	Firmicutes; Clostridia; Clostridiales; Clostridiaceae; Clostridium;	0.6
517	Firmicutes; Clostridia; Clostridiales; Veillonellaceae; Dialister;	0.6
493	Firmicutes; Clostridia; Clostridiales; Ruminococcaceae; ;	0.6
490	Firmicutes; Clostridia; Clostridiales; Ruminococcaceae; Ruminococcus;	0.6
483	Bacteroidetes; Bacteroidia; Bacteroidales; Prevotellaceae; Prevotella;	0.6
478	Firmicutes; Clostridia; Clostridiales; [Mogibacteriaceae]; ;	0.6

ANCOM: Analysis of Composition of Microbiomes; BMI: body mass index

Supplementary table 5: PERMANOVA results- reduced model (n=176)						
(A) Jensen Shannon Divergence						
	Df	SumsOfSqs	MeanSqs	F.Model	R2	Pr(>F)
Crowding index	1	1.372	1.372	14.423	0.072	0.001
Age, years	1	0.211	0.211	2.218	0.011	0.031
Sex	1	0.139	0.139	1.458	0.007	0.133
Village	2	1.262	0.631	6.636	0.066	0.001
Residuals	170	16.167	0.095	NA	0.844	NA
Total	175	19.150	NA	NA	1.000	NA
(B) Weighted UniFrac						
	Df	SumsOfSqs	MeanSqs	F.Model	R2	Pr(>F)
Crowding index	1	1.177	1.177	10.047	0.052	0.001
Age, years	1	0.272	0.272	2.321	0.012	0.010
Sex	1	0.149	0.149	1.274	0.007	0.166
Village	2	1.064	0.532	4.539	0.047	0.001
Residuals	170	19.919	0.117	NA	0.882	NA
Total	175	22.581	NA	NA	1.000	NA
DF: degrees of freedom; NA: not applicable; PERMANOVA: Permutational multivariate analysis of variance						

Supplementary table 6 - ANCOM results adjusted for age, sex and village (n=176)		
W_stat	Taxonomy	etection Level
761	Bacteroidetes; Bacteroidia; Bacteroidales; Rikenellaceae; Alistipes; onderdonkii	0.9
760	Bacteroidetes; Bacteroidia; Bacteroidales; Bacteroidaceae; Bacteroides; uniformis	0.9
759	Bacteroidetes; Bacteroidia; Bacteroidales; Prevotellaceae; Prevotella; stercorea	0.9
751	Firmicutes; Clostridia; Clostridiales; Veillonellaceae; Phascolarctobacterium;	0.9
710	Bacteroidetes; Bacteroidia; Bacteroidales; Rikenellaceae; Alistipes; putredinis	0.9
695	Firmicutes; Clostridia; Clostridiales; Ruminococcaceae; Faecalibacterium; prausnitzii	0.8
681	Firmicutes; Clostridia; Clostridiales; Lachnospiraceae; ;	0.8
653	Bacteroidetes; Bacteroidia; Bacteroidales; Prevotellaceae; Prevotella; copri	0.8
652	Bacteroidetes; Bacteroidia; Bacteroidales; Porphyromonadaceae; Parabacteroides;	0.8
634	Bacteroidetes; Bacteroidia; Bacteroidales; Bacteroidaceae; Bacteroides; NA	0.8
599	Bacteroidetes; Bacteroidia; Bacteroidales; Prevotellaceae; ;	0.7
586	Bacteroidetes; Bacteroidia; Bacteroidales; [Paraprevotellaceae]; Paraprevotella;	0.7
584	Firmicutes; Clostridia; Clostridiales; Ruminococcaceae; NA; NA	0.7
560	Firmicutes; Clostridia; Clostridiales; NA; NA; NA	0.7
558	Bacteroidetes; Bacteroidia; Bacteroidales; Bacteroidaceae; Bacteroides; caccae	0.7
555	Firmicutes; Clostridia; Clostridiales; Lachnospiraceae; ;	0.7
554	Firmicutes; Clostridia; Clostridiales; Lachnospiraceae; Coprococcus; eutactus	0.7
546	Bacteroidetes; Bacteroidia; Bacteroidales; [Paraprevotellaceae]; [Prevotella];	0.7
543	Bacteroidetes; Bacteroidia; Bacteroidales; Porphyromonadaceae; Parabacteroides; distasonis	0.6
535	Firmicutes; Clostridia; Clostridiales; ; ;	0.6
532	Firmicutes; Erysipelotrichi; Erysipelotrichales; Erysipelotrichaceae; Bulleidia; p-1630-c5	0.6
529	Firmicutes; Clostridia; Clostridiales; ; ;	0.6
499	Tenericutes; Mollicutes; RF39; ; ;	0.6
494	Firmicutes; Clostridia; Clostridiales; Ruminococcaceae; NA; NA	0.6
488	Firmicutes; Clostridia; Clostridiales; Lachnospiraceae; Coprococcus;	0.6
487	Firmicutes; Clostridia; Clostridiales; Ruminococcaceae; ;	0.6
482	Bacteroidetes; Bacteroidia; Bacteroidales; ; ;	0.6
471	Firmicutes; Erysipelotrichi; Erysipelotrichales; Erysipelotrichaceae; ;	0.6

ANCOM: Analysis of Composition of Microbiomes; BMI: body mass index

Supplementary table 7 - ANCOM results adjusted for age, sex and stratified by village			
Village	W_stat	Taxonomy	Detection Level
**Villages A & B (n=106)**	722	Bacteroidetes; Bacteroidia; Bacteroidales; Prevotellaceae; Prevotella; stercorea	0.9
	718	Firmicutes; Clostridia; Clostridiales; Veillonellaceae; Phascolarctobacterium;	0.9
	657	Bacteroidetes; Bacteroidia; Bacteroidales; Rikenellaceae; Alistipes; onderdonkii	0.8
	621	Bacteroidetes; Bacteroidia; Bacteroidales; [Paraprevotellaceae]; Paraprevotella;	0.8
	580	Bacteroidetes; Bacteroidia; Bacteroidales; Prevotellaceae; Prevotella; copri	0.7
	572	Bacteroidetes; Bacteroidia; Bacteroidales; Bacteroidaceae; Bacteroides; uniformis	0.7
	519	Firmicutes; Clostridia; Clostridiales; Lachnospiraceae; NA; NA	0.6
	453	Firmicutes; Clostridia; Clostridiales; Lachnospiraceae; Coprococcus;	0.6
**Village C (n=70)**	707	Bacteroidetes; Bacteroidia; Bacteroidales; Bacteroidaceae; Bacteroides; uniformis	0.9
	680	Firmicutes; Clostridia; Clostridiales; Lachnospiraceae; ;	0.8
	668	Bacteroidetes; Bacteroidia; Bacteroidales; Rikenellaceae; ;	0.8
	663	Bacteroidetes; Bacteroidia; Bacteroidales; Porphyromonadaceae; Parabacteroides;	0.8
	620	Bacteroidetes; Bacteroidia; Bacteroidales; Bacteroidaceae; Bacteroides; NA	0.8
	611	Firmicutes; Clostridia; Clostridiales; Ruminococcaceae; NA; NA	0.8
	605	Bacteroidetes; Bacteroidia; Bacteroidales; Bacteroidaceae; Bacteroides; caccae	0.8
	566	Bacteroidetes; Bacteroidia; Bacteroidales; Rikenellaceae; Alistipes; onderdonkii	0.7
	557	Proteobacteria; Betaproteobacteria; Burkholderiales; Alcaligenaceae; Sutterella;	0.7
	543	Firmicutes; Clostridia; Clostridiales; Lachnospiraceae; ;	0.7
	520	Firmicutes; Clostridia; Clostridiales; Ruminococcaceae; Oscillospira;	0.6
	496	Firmicutes; Clostridia; Clostridiales; Ruminococcaceae; Faecalibacterium; prausnitzii	0.6
	488	Firmicutes; Clostridia; Clostridiales; Lachnospiraceae; ;	0.6

ANCOM: Analysis of Composition of Microbiomes; BMI: body mass index

Supplementary table 8 - PERMANOVA subsampled (n=141)					
(A) Jensen Shannon Divergence PERMANOVA					
	**Df**	**SumOfSqs**	**R2**	**F**	**Pr(>F)**
**Village**	1	1.531	0.101	16.036	0.001
**Age, years**	1	0.150	0.010	1.569	0.068
**Sex**	1	0.150	0.010	1.567	0.087
**Household crowding**	1	0.449	0.029	4.702	0.001
**Mother's education, years**	1	0.062	0.004	0.651	0.849
**BMI Z score**	1	0.086	0.006	0.896	0.523
**Residual**	134	12.797	0.841	NA	NA
**Total**	140	15.225	1.000	NA	NA
(B) Weighted UniFrac PERMANOVA					
	**Df**	**SumOfSqs**	**R2**	**F**	**Pr(>F)**
**Village**	1	1.565	0.080	12.415	0.001
**Age, years**	1	0.251	0.013	1.990	0.019
**Sex**	1	0.142	0.007	1.123	0.300
**Household crowding**	1	0.497	0.025	3.943	0.001
**Mother's education, years**	1	0.078	0.004	0.615	0.908
**BMI Z score**	1	0.108	0.006	0.860	0.590
**Residual**	134	16.893	0.865	NA	NA
**Total**	140	19.534	1.000	NA	NA
BMI: Body mass index; DF: degrees of freedom; NA: not applicable; PERMANOVA: Permutational multivariate analysis of variance					

Supplementary table 9 - ANCOM - subsampled (n=141), adjusted for age, sex, household crowding index, BMI and mother's education level		
W_stat	Taxonomy	Detection Level
746	Bacteroidetes; Bacteroidia; Bacteroidales; Prevotellaceae; Prevotella; copri	0.9
745	Bacteroidetes; Bacteroidia; Bacteroidales; Prevotellaceae; Prevotella; copri	0.9
739	Firmicutes; Clostridia; Clostridiales; Veillonellaceae; Dialister;	0.9
722	Bacteroidetes; Bacteroidia; Bacteroidales; Rikenellaceae; Alistipes; putredinis	0.9
716	Actinobacteria; Actinobacteria; Bifidobacteriales; Bifidobacteriaceae; Bifidobacterium;	0.9
705	Firmicutes; Erysipelotrichi; Erysipelotrichales; Erysipelotrichaceae; [Eubacterium]; biforme	0.9
701	Firmicutes; Erysipelotrichi; Erysipelotrichales; Erysipelotrichaceae; [Eubacterium]; biforme	0.9
669	Firmicutes; Clostridia; Clostridiales; Ruminococcaceae; Oscillospira;	0.8
667	Firmicutes; Clostridia; Clostridiales; Ruminococcaceae; NA; NA	0.8
665	Firmicutes; Clostridia; Clostridiales; Ruminococcaceae; Faecalibacterium; prausnitzii	0.8
631	Firmicutes; Clostridia; Clostridiales; Ruminococcaceae; Ruminococcus;	0.8
630	Proteobacteria; Betaproteobacteria; Burkholderiales; Alcaligenaceae; Sutterella;	0.8
625	Firmicutes; Clostridia; Clostridiales; [Mogibacteriaceae]; ;	0.8
623	Bacteroidetes; Bacteroidia; Bacteroidales; Prevotellaceae; Prevotella;	0.8
593	Firmicutes; Clostridia; Clostridiales; Clostridiaceae; Clostridium;	0.7
586	Firmicutes; Clostridia; Clostridiales; Veillonellaceae; Dialister;	0.7
581	Firmicutes; Clostridia; Clostridiales; ; ;	0.7
577	Firmicutes; Erysipelotrichi; Erysipelotrichales; Erysipelotrichaceae; Catenibacterium;	0.7
573	Proteobacteria; Betaproteobacteria; Burkholderiales; Alcaligenaceae; Sutterella;	0.7
543	Proteobacteria; Betaproteobacteria; Burkholderiales; Alcaligenaceae; Sutterella;	0.7
542	Bacteroidetes; Bacteroidia; Bacteroidales; Prevotellaceae; Prevotella;	0.7
538	Firmicutes; Clostridia; Clostridiales; [Mogibacteriaceae]; ;	0.7
537	Firmicutes; Clostridia; Clostridiales; Ruminococcaceae; Oscillospira;	0.7
534	Bacteroidetes; Bacteroidia; Bacteroidales; Bacteroidaceae; Bacteroides; ovatus	0.7
532	Bacteroidetes; Bacteroidia; Bacteroidales; Prevotellaceae; Prevotella;	0.7
530	Firmicutes; Clostridia; Clostridiales; Lachnospiraceae; ;	0.7
530	Firmicutes; Clostridia; Clostridiales; Ruminococcaceae; NA; NA	0.7
510	Firmicutes; Clostridia; Clostridiales; Ruminococcaceae; Oscillospira;	0.6
506	Bacteroidetes; Bacteroidia; Bacteroidales; [Barnesiellaceae]; ;	0.6
502	Firmicutes; Clostridia; Clostridiales; Ruminococcaceae; ;	0.6
495	Bacteroidetes; Bacteroidia; Bacteroidales; Prevotellaceae; Prevotella; stercorea	0.6
477	Firmicutes; Clostridia; Clostridiales; Ruminococcaceae; Oscillospira;	0.6
470	Firmicutes; Clostridia; Clostridiales; Ruminococcaceae; Ruminococcus;	0.6
469	Firmicutes; Clostridia; Clostridiales; Ruminococcaceae; NA; NA	0.6
450	Firmicutes; Clostridia; Clostridiales; Ruminococcaceae; Oscillospira;	0.6

ANCOM: Analysis of Composition of Microbiomes; BMI: body mass index

Supplementary table 10 - PERMANOVA subsampled (n=141)					
(A) Jensen Shannon Divergence PERMANOVA					
	**Df**	**SumOfSqs**	**R2**	**F**	**Pr(>F)**
**Household crowding**	1	1.228	0.08	12.882	0.001
**Village**	2	0.972	0.064	5.098	0.001
**Sex**	1	0.144	0.009	1.508	0.1
**Age**	1	0.072	0.005	0.75	0.736
**Residual**	135	12.872	0.842	NA	NA
**Total**	140	15.288	1	NA	NA
(B) Weighted UniFrac PERMANOVA					
	**Df**	**SumOfSqs**	**R2**	**F**	**Pr(>F)**
**Household crowding**	1	1.048	0.06	9.213	0.001
**Village**	2	0.919	0.052	4.037	0.001
**Sex**	1	0.124	0.007	1.092	0.294
**Age**	1	0.085	0.005	0.747	0.783
**Residual**	135	15.36	0.876	NA	NA
**Total**	140	17.537	1	NA	NA
DF: degrees of freedom; NA: not applicable; PERMANOVA: Permutational multivariate analysis of variance					

Supplementary table 11 - ANCOM - subsampled (n=141), adjusted for village, age, sex, BMI and mother's education level

W_stat	Taxonomy	Detection Level
734	Bacteroidetes; Bacteroidia; Bacteroidales; Prevotellaceae; Prevotella; stercorea	0.9
728	Bacteroidetes; Bacteroidia; Bacteroidales; Rikenellaceae; Alistipes; onderdonkii	0.9
724	Bacteroidetes; Bacteroidia; Bacteroidales; Bacteroidaceae; Bacteroides; uniformis	0.9
711	Firmicutes; Clostridia; Clostridiales; Veillonellaceae; Phascolarctobacterium;	0.9
695	Bacteroidetes; Bacteroidia; Bacteroidales; Rikenellaceae; Alistipes; putredinis	0.9
676	Bacteroidetes; Bacteroidia; Bacteroidales; [Paraprevotellaceae]; [Prevotella];	0.9
675	Bacteroidetes; Bacteroidia; Bacteroidales; Bacteroidaceae; Bacteroides; caccae	0.9
660	Bacteroidetes; Bacteroidia; Bacteroidales; Prevotellaceae; Prevotella; copri	0.8
636	Bacteroidetes; Bacteroidia; Bacteroidales; [Paraprevotellaceae]; Paraprevotella;	0.8
631	Bacteroidetes; Bacteroidia; Bacteroidales; Bacteroidaceae; Bacteroides; NA	0.8
623	Firmicutes; Clostridia; Clostridiales; NA; NA; NA	0.8
597	Firmicutes; Clostridia; Clostridiales; Lachnospiraceae; ;	0.7
592	Firmicutes; Clostridia; Clostridiales; Ruminococcaceae; Faecalibacterium; prausnitzii	0.7
578	Firmicutes; Clostridia; Clostridiales; Ruminococcaceae; NA; NA	0.7
564	Firmicutes; Clostridia; Clostridiales; Lachnospiraceae; ;	0.7
564	Firmicutes; Clostridia; Clostridiales; Ruminococcaceae; ;	0.7
561	Bacteroidetes; Bacteroidia; Bacteroidales; Porphyromonadaceae; Parabacteroides;	0.7
549	Firmicutes; Clostridia; Clostridiales; Ruminococcaceae; NA; NA	0.7
548	Firmicutes; Clostridia; Clostridiales; ; ;	0.7
545	Firmicutes; Clostridia; Clostridiales; ; ;	0.7
543	Tenericutes; Mollicutes; RF39; ; ;	0.7
523	Actinobacteria; Actinobacteria; Bifidobacteriales; Bifidobacteriaceae; Bifidobacterium; bifidum	0.7
517	Firmicutes; Erysipelotrichi; Erysipelotrichales; Erysipelotrichaceae; ;	0.6
512	Firmicutes; Clostridia; Clostridiales; Veillonellaceae; Dialister;	0.6
500	Bacteroidetes; Bacteroidia; Bacteroidales; Porphyromonadaceae; Parabacteroides; distasonis	0.6
473	Firmicutes; Clostridia; Clostridiales; Lachnospiraceae; ;	0.6
465	Bacteroidetes; Bacteroidia; Bacteroidales; Prevotellaceae; ;	0.6
456	Lentisphaerae; [Lentisphaeria]; Victivallales; Victivallaceae; ;	0.6

ANCOM: Analysis of Composition of Microbiomes; BMI: body mass index
